# 3D kinematics using dual quaternions: theory and applications in neuroscience

**DOI:** 10.3389/fnbeh.2013.00007

**Published:** 2013-02-20

**Authors:** Guillaume Leclercq, Philippe Lefèvre, Gunnar Blohm

**Affiliations:** ^1^Institute of Information and Communication Technologies, Electronics and Applied Mathematics, Université Catholique de LouvainLouvain-la-Neuve, Belgium; ^2^Institute of Neuroscience, Université Catholique de LouvainBrussels, Belgium; ^3^Centre for Neuroscience Studies, Queen's UniversityKingston, ON, Canada; ^4^Canadian Action and Perception NetworkToronto, ON, Canada

**Keywords:** geometric algebra, forward kinematics, inverse kinematics, reference frame transformation

## Abstract

In behavioral neuroscience, many experiments are developed in 1 or 2 spatial dimensions, but when scientists tackle problems in 3-dimensions (3D), they often face problems or new challenges. Results obtained for lower dimensions are not always extendable in 3D. In motor planning of eye, gaze or arm movements, or sensorimotor transformation problems, the 3D kinematics of external (stimuli) or internal (body parts) must often be considered: how to describe the 3D position and orientation of these objects and link them together? We describe how dual quaternions provide a convenient way to describe the 3D kinematics for position only (point transformation) or for combined position and orientation (through line transformation), easily modeling rotations, translations or screw motions or combinations of these. We also derive expressions for the velocities of points and lines as well as the transformation velocities. Then, we apply these tools to a motor planning task for manual tracking and to the modeling of forward and inverse kinematics of a seven-dof three-link arm to show the interest of dual quaternions as a tool to build models for these kinds of applications.

## 1. Introduction

Our environment is 3-dimensional (3D) and our body can be represented as a rigid multibody system evolving in that 3D environment, with different body parts moving relative to each other. Accurately describing the 3D kinematics of such systems is important for diverse applications in neuroscience which involve 3D kinematics: (1) forward kinematics of different 3D systems: the position and/or orientation of an end-effector (a tool held by the hand for example), (2) 3D Reference frame transformation or reference frame encoding [for example: 3D vestibulor-ocular reflex (VOR), 3D visuomotor transformation, 3D eye-head gaze shifts, 3D spatial updating, …], and (3) inverse kinematics: the set of joint angles corresponding to a given end-effector position and velocity. In practice, these models allow us to analyze 3D data acquired through behavioral experiments as well as to make predictions.

To model the kinematics of a rigid multibody system, we use the framework of the geometric algebra introduced by David Hestenes (see the textbook Hestenes, 1986), which easily applies to and is very convenient to model mechanical systems, in particular the kinematics. Hestenes applied this approach to some eye (Hestenes, 1994a) and arm reaching (Hestenes, 1994b) movements. Actually, he chose the geometric algebra of quaternions which have been sometimes used to model 3D eye, head and arm movements (see for example: (Tweed and Vilis, 1987; Straumann et al., 1991; Hore et al., 1992; Haslwanter, 1995; Crawford and Guitton, 1997). Quaternions allow us to easily deal with rotations but not with translations. Yet we have to deal with translations since the origins of reference frames attached to each body generally do not coincide. For example, the eye rotation center is offset from the head rotation center. In order to deal with translations, in addition to the rotation, we will use the geometric algebra of dual quaternions (see Bayro-Corrochano, 2003). Dual quaternions also allow to elegantly represent a screw motion (Funda and Paul, 1990b; Aspragathos and Dimitros, 1998), defined as a combined rotation and translation along the rotation axis. They have been used in Blohm and Crawford (2007) to model combined 3D eye and head rotations in the context of 3D visuomotor transformation for reaching movements. While dual quaternions have not been widely used in the neuroscience community, they have been applied in other fields. Indeed, they have been employed with success in computer vision (Walker et al., 1991; Phong et al., 1993; Goddard and Abidi, 1998; Torsello et al., 2011) or in robotics (Daniilidis, 1999; Bayro-Corrochano et al., 2000).

The goal of this paper is to provide a tutorial of the dual quaternion geometric algebra to the neuroscientists and then to show its interests and advantages to several applications in sensorimotor control. First, we summarize the theory necessary to introduce the different applications. The different dual quaternion operations are described and we also provide our MATLAB implementation of these operations in supplementary materials for the potential interested reader. Then we describe several applications using the dual quaternion formalism.

There are several reasons to use dual quaternions to represent the 3D kinematics. First, it is a compact and geometrically meaningful way of representing 3D rotations, translations and screw motions (Funda and Paul, 1990a; Kim and Kumar, 1990; Aspragathos and Dimitros, 1998; Daniilidis, 1999; Kavan et al., 2008). Furthermore, there are no singularities when representing 3D rotations (this is not the case if a Fick, Helmoltz or Euler representation is chosen), because a rotation is represented through a single angle θ and the axis of rotation **n** (Chou, 1992; Grassia, 1998). Moreover computational requirements (in terms of storage and speed) are lower than for other methods, including homogeneous coordinates (Funda et al., 1990; Funda and Paul, 1990b; Walker et al., 1991; Aspragathos and Dimitros, 1998), which could be important for behavioral experiments needing real-time data and processing (for example, a forward kinematic model applied to on-line raw data) but also for post-processing treatments in data analysis and model predictions. In an implementation perspective again, the neuroscience researcher concerned with the practical implementation of the transformations using dual quaternions will most likely want to consider many transformations simultaneously. For example, when analyzing experimental data, or when making predictions (from a few thousand to many more). In this context, dual quaternions are much more convenient than homogenous matrices for example. Indeed, we easily deal with an arbitrary number N of simultaneous dual quaternions transformations (using standard matrix operations), while for homogeneous coordinates, it requires the use of 3D tensors, which is possible but not easy to implement. Yet neuroscience researchers want a tool efficient and easy to use at the same time. Last but not least the dual quaternion formalism can be used to model point and lines transformations (Bayro-Corrochano, 2003), which is not the case for other formalisms like homogeneous coordinates (only point transformations). Yet applications sometimes demand that we combine both types of transformations (see section 3.3 of this article) and dual quaternions provide a unified and easy way to do that. We would have had to employ two different approaches to perform kinematic operations on points and lines if we had not used dual quaternions.

## 2. Theory

In this section, we provide a tutorial or short description of the dual quaternion algebra based on the literature (Hestenes, 1994a; Bayro-Corrochano, 2003). The reader familiar with these concepts can skip this part and move to section 3. However, as far as we know of, many readers in the sensorimotor science community are not familiar with dual quaternions and therefore they will find in this section many useful dual quaternions operations which can be useful to model their 3D problem in neuroscience. Moreover, we provide a MATLAB implementation for each of these operations, for the reader who would like to use dual quaternions but who perhaps would be restrained by the implementation. This toolbox is available for download at the followings urls:

- http://www.compneurosci.com/doc/DualQuaternionToolbox.zip
- On the File exchange of Matlab Central: http://www.mathworks.com/matlabcentral/fileexchange/39288. Users are able to provide feedback, comment and rate the files.

In the following, we introduce the dual quaternion algebra, based on the quaternion algebra. Then we introduce the rotation and translation operations using the dual quaternion algebra. Afterwards we describe how points and lines can be encoded using dual quaternions, before explaining how applying the rotation or translation on these objects. Then we describe what a screw motion is and how it is encoded using a dual quaternion. Finally we briefly talk about the implementation and conversion to other formalisms.

### 2.1. Basics

#### 2.1.1. Quaternion geometric algebra

First let us define the geometric product of two 3D vectors $\vec{a}$ and $\vec{b}$ (see Hestenes, 1994a; Bayro-Corrochano, 2003). It is denoted $\vec{a}\vec{b}$ and can be expressed as:

(1) $$\vec{a}\vec{b}\;\dot{=}\;\vec{a}\cdot \vec{b}+\vec{a}\wedge \vec{b}$$

where $\vec{a}\;\cdot \;\vec{b}$ is the classical dot product yielding a scalar number and $\vec{a}\wedge \vec{b}$ is a new entity called a bivector, resulting from the wedge product of $\vec{a}$ and $\vec{b}$. This wedge product is antisymmetric: $\vec{a}\wedge \vec{b}=-\vec{b}\wedge \vec{a}$. We will see later how to compute it in practice. Now let us consider three orthonormal basis vectors in a right-handed reference frame, $\vec{\sigma_{1}}$, $\vec{\sigma_{2}}$, and $\vec{\sigma_{3}}$. From the definition of the geometric product and the antisymmetric property of the wedge product, we can get that:

$$\vec{\sigma_{i}}\vec{\sigma_{j}}=\left\{ \begin{array}{ccc} 1 & \text{if} & i=j\\ \vec{\sigma_{i}}\wedge \vec{\sigma_{j}} & \text{if} & i\neq j \end{array}\right.$$

Therefore, we obtain three basis bivectors: $\vec{\sigma_{1}}\vec{\sigma_{2}}$, $\vec{\sigma_{2}}\vec{\sigma_{3}}$, and $\vec{\sigma_{3}}\vec{\sigma_{1}}$. Then the *unit right-handed pseudoscalar i* is defined (see Hestenes, 1994a) as:

$$i=\vec{\sigma_{1}}\vec{\sigma_{2}}\vec{\sigma_{3}}=\vec{\sigma_{1}}\wedge \vec{\sigma_{2}}\wedge \vec{\sigma_{3}}$$

*i* is thus an entity which is called a *trivector*. The *i* symbol is used by analogy with the complex numbers. Indeed, using the properties of the geometric product and the definition of *i*, we have: *i*^2^ = −1. *i* is a duality operator since we can notice that:

$$\begin{array}{l} \vec{\sigma_{1}}\vec{\sigma_{2}}=i\vec{\sigma_{3}}\\ \vec{\sigma_{2}}\vec{\sigma_{3}}=i\vec{\sigma_{1}}\\ \vec{\sigma_{3}}\vec{\sigma_{1}}=i\vec{\sigma_{2}} \end{array}$$

Therefore, the three basis bivectors are dual entities to the three basis vectors and vice-versa, which implies that for every bivector **a**, there exists a corresponding vector $\vec{a}$ such that $a=i\vec{a}$. In the following, we will always use the bold notation for bivectors, and top arrows for vectors. In particular, the bivector obtained by the wedge product can be expressed as:

$$\vec{a}\wedge \vec{b}=i(\vec{a}\times \vec{b})$$

where $\vec{a}\times \vec{b}$ is a vector representing the classical cross product of the vectors $\vec{a}$ and $\vec{b}$. A quaternion is the sum of a scalar and a bivector (Hestenes, 1994a):

$$Q=q_{0}+q=q_{0}+i\vec{q}$$

and can be obtained as the geometric product of two vectors, as shown by Equation (1). The sum of two quaternions $A=a_{0}+i\vec{a}$ and $B=b_{0}+i\vec{b}$ is a quaternion $C=c_{0}+i\vec{c}$ computed as follows:

$$C=A+B=(\underset{c_{0}}{\underset{︸}{a_{0}+b_{0}}})+i(\underset{\vec{c}}{\underset{︸}{\vec{a}+\vec{b}}})$$

The multiplication of two quaternions $A=a_{0}+i\vec{a}$ and $B=b_{0}+i\vec{b}$ is a quaternion $C=c_{0}+i\vec{c}$. It is computed as (see details in Appendix A):

$$\begin{array}{l} C=AB=(a_{0}+i\vec{a})(b_{0}+i\vec{b})=\underset{c_{0}}{\underset{︸}{\left(a_{0}b_{0}-\vec{a}\cdot \vec{b}\right)}}\\ \;+i\underset{\vec{c}}{\underset{︸}{\left(a_{0}\vec{b}+b_{0}\vec{a}-\vec{a}\times \vec{b}\right)}} \end{array}$$

Other quaternion operations are also useful. The conjugate of a quaternion *A* is defined by:

$$A^{*}={\left(a_{0}+i\vec{a}\right)}^{*}=a_{0}-i\vec{a}$$

The norm of a quaternion *A* is denoted: $||A||=\sqrt{\left(a_{0}^{2}+\vec{a}\;\cdot \;\vec{a}\right)}$. The inverse of a quaternion *A* is denoted *A*^−1^ and defined as $A^{-1}=\frac{A^{*}}{||A|{|}^{2}}$.

#### 2.1.2. Dual quaternion geometric algebra

A dual quaternion *D* is defined by

$$D\;\dot{=}\;D_{0}+\epsilon D_{1}$$

where *D*_0_ and *D*_1_ are two quaternions and ϵ is a mathematical operator with the property that:

(2) $$\epsilon^{2}=0$$

The multiplication of two dual quaternions *D* = *D*_0_ + ϵ*D*_1_ and *E* = *E*_0_ + ϵ*E*_1_ is another dual quaternion *F* = *F*_0_ + ϵ*F*_1_ and using the property (2), *F* is equal to:

$$F=DE=(D_{0}+\epsilon D_{1})(E_{0}+\epsilon E_{1})=\underset{F_{0}}{\underset{︸}{D_{0}E_{0}}}\;+\;\epsilon (\underset{F_{1}}{\underset{︸}{D_{0}E_{1}+D_{1}E_{0}}})$$

We notice that the dual quaternion multiplication involves three quaternion multiplications.

Several conjugates exist for the dual quaternion *D* = *D*_0_ + ϵ*D*_1_, which are used depending on the operations needed. The first one is obtained by applying the quaternion conjugate to each quaternion composing the dual quaternion.

$$D^{*}=D_{0}^{*}+\epsilon D_{1}^{*}$$

The conjugate of a dual number can also be used:

$$\bar{D}=D_{0}-\epsilon D_{1}$$

And both operations can also be combined:

$$\bar{D^{*}}=D_{0}^{*}-\epsilon D_{1}^{*}$$

The following identities are also useful:

$$\begin{array}{l} {\left(AB\right)}^{*}=B^{*}A^{*}\\ \;\left(\bar{AB}\right)=\bar{A}\bar{B}\\ {\left(\bar{AB}\right)}^{*}={\bar{B}}^{*}{\bar{A}}^{*} \end{array}$$

The norm of a dual quaternion *D* is a dual number (a dual number is of the form *a* = *a*_0_ + ϵ*a*_1_ where *a*_0_ and *a*_1_ are real scalar numbers and ϵ is the same operator as for dual quaternions, with the property that ϵ^2^ = 0). The dual quaternion norm is computed in the following way (see Kavan et al., 2008):

$$||D||=||D_{0}||+\epsilon {\left[\frac{D_{0}^{*}D_{1}+D_{1}^{*}D_{0}}{2||D_{0}||}\right]}_{\text{scalar}}$$

where ||*D*_0_|| is the quaternion norm of *D*_0_ and [*Q*]_scalar_ is the scalar part *q*_0_ of a quaternion *Q* = *q*_0_ + **q**. The inverse of a dual quaternion *A* = *A*_0_ + ϵ*A*_1_ can be computed and is written (see Kavan et al., 2008):

$$A^{-1}=\frac{A^{*}}{||A|{|}^{2}}$$

If the non-dual part, *A*_0_, of the dual quaternion *A* has a zero norm, then *A* has no inverse.

In the next sections, the dual quaternions representing rotations, translations and or screw motions, as well as points and lines, are described using unitary dual quaternions, i.e., dual quaternions with a norm equal to 1. They are unitary under the condition that:

$$\begin{array}{r} ||D_{0}||=1\\ D_{0}^{*}D_{1}+D_{1}^{*}D_{0}=0 \end{array}$$

Therefore, unit dual quaternions belong to a 6-dimensional manifold and are specified by six different parameters. From now on, we always use the bivector notation in order to avoid writing the *i* symbol at each equation. Anyway, remember that each bivector **a** is directly related to its counterpart vector $\vec{a}$ by the relation $a=i\vec{a}$.

### 2.2. Rotations and translations

In this section, we describe the dual quaternions associated with rotations and translations, as well as their velocity: rotational velocity, translational velocity. These operators will be used and combined later to carry out complex kinematic transformations.

#### 2.2.1. Rotation

By Euler's theorem, any 3D rotation may be described by a unique unitary rotation axis **n** and a single rotation angle θ. In the dual quaternion framework, a pure rotation (whose rotation axis goes through the origin of the reference frame) is described by:

(3) Rrot=cos(θ2)+n sin(θ2)

where **n** is a bivector representing the unitary rotation axis coordinates expressed in the reference frame and θ is the rotation angle. Actually, *R*_rot_ is a quaternion, or a dual quaternion with a dual part (the part premultiplied by ϵ) equal to 0.

#### 2.2.2. Translation

A pure translation is defined by its unitary axis **t** and its distance *d*. A translation dual quaternion *T*_trans_ is written under the following form:

$$T_{\text{trans}}=1+\epsilon \frac{d}{2}t$$

#### 2.2.3. Rotational velocity

Potential users should pay attention to the fact that in 3-D, the rotational velocity quaternion is not the derivative of the rotation quaternion (this is also the case if classical rotation matrices are considered). It has been shown by Hestenes (1986) that the derivative of the rotation quaternion with respect to time can be written as:

(4) $${\dot{R}}_{\text{rot}}=\frac{1}{2}R_{\text{rot}}\Omega_{\text{rot}}$$

where $\Omega_{\text{rot}}=\Omega_{rot}=i\Omega_{\text{rot}}^{\to }$ is a rotational velocity dual quaternion which has a dual part equal to zero, and which has a non-dual part with a zero scalar component. Ω_rot_ represents the *rotational velocity*. Kinematically, the norm of Ω_**rot**_, $\left||\Omega_{\text{rot}}^{\to }|\right|$ represents the *instantaneous* angular velocity while the normalized vector $\frac{\vec{\Omega_{\text{rot}}}}{\left||\vec{\Omega_{\text{rot}}}|\right|}$ represents the *instantaneous* rotation axis. Also, it can be shown that:

$$\frac{d(R_{\text{rot}}^{*})}{dt}={\left(\frac{dR_{\text{rot}}}{dt}\right)}^{*}=-\frac{1}{2}\Omega_{rot}R_{\text{rot}}^{*}$$

#### 2.2.4. Translational velocity

The translational velocity dual quaternion is derived from the translation dual quaternion *T*_trans_. Differentiating the expression of the translation dual quaternion *T*_trans_, we obtain:

$$\begin{array}{l} {\dot{T}}_{\text{trans}}=0+\epsilon \left(\frac{\dot{d}(t)}{2}t(t)+\frac{d(t)}{2}\dot{t}(t)\right)\\ \;=\epsilon \frac{v}{2} \end{array}$$

where $v\dot{=}\dot{d}(t)t(t)+d(t)\dot{t}(t)$.

### 2.3. Description of point and line positions and velocities

Here we describe how we can describe either points (location and velocity) or lines (location and velocity) using the dual quaternion formalism.

#### 2.3.1. Point position and velocity

Following what is done by Bayro-Corrochano (2003) for example, a 3D physical point P with coordinates **x** in a given reference frame R is represented by the dual quaternion *X* = 1 + ϵ**x**.

The velocity **ẋ** of point P is represented by the dual quaternion *V* = *Ẋ* = ϵ**ẋ**.

#### 2.3.2. Line position and velocity

Dual quaternions provide a convenient way to represent lines (Daniilidis, 1999; Bayro-Corrochano, 2003). A line can be represented by six coordinates (Plücker coordinates), specifying the line orientation **n** and the position of an arbitrary point of the line in space, **p**. The line representation is moreover independent of the choice of the point **p**. A line dual quaternion *L* is written:

(5) $$L=n+\epsilon \underset{\dot{=}m}{\underset{︸}{\vec{p}\wedge \vec{n}}}$$

We can notice that **m** is indeed independent of the choice of **p** since the cross product $\vec{p}\times \vec{n}$ depends only on the component of $\vec{p}$ orthogonal to $\vec{n}$, and this orthogonal component is obviously the same for every point on the line *L*. Note also that a line dual quaternion is unitary.

The line velocity, $\dot{L}$, is obtained by computing the derivative of the line dual quaternion *L*:

(6) $$\begin{array}{l} \dot{L}=\dot{n}+\epsilon \dot{m}\\ \;=\dot{n}+\epsilon \frac{d(\vec{p}\wedge \vec{n})}{dt}\\ \;=\dot{n}+\epsilon \left(\dot{\vec{p}}\wedge \vec{n}+\vec{p}\wedge \dot{\vec{n}}\right) \end{array}$$

We see that the line velocity dual quaternion is related to the rate of change of $\vec{n}$, as well as the rate of change of $\vec{p}$. Note that Equation (6) does not depend on the arbitrary choice of $\vec{p}$ and its rate of change (see proof in Appendix B).

### 2.4. Kinematic operations on points and lines

Now we describe the kinematic transformations on point and lines, as the dual quaternion formalism allows us to use the same kinematic operators for point or line transformations, which is a major advantage.

Let us consider the reference frame R′, with the same origin as reference frame R, but lying in a different orientation. The transformation between both reference frames is a pure rotation with axis **n** and angle θ. Then, a point P has the following coordinates in the R′ frame (passive rotation):

(7) $$X^{\prime }=R_{\text{rot}}X{\bar{R_{\text{rot}}}}^{*}=1+\epsilon \left(R_{\text{rot}}x{\bar{R_{\text{rot}}}}^{*}\right)$$

where *R*_rot_ is the rotation quaternion. ${\bar{R_{\text{rot}}}}^{*}$ is the conjugate quaternion: Rrot¯*=cos(θ2)−n sin(θ2). For rotations dual quaternions, ${\bar{R_{\text{rot}}}}^{*}=R_{\text{rot}}^{*}$, since rotation dual quaternions have a dual component equal to zero. Therefore, in the following, we use R^*^_rot_ instead of ${\bar{R_{\text{rot}}}}^{*}$ since it is shorter to write.

From another point of view, we may want to know the new position of a mobile point *P* after a rotation *R*_rot_ applied to a reference position *P*_0_ = 1 + ϵ**x**_0_ in a given reference frame R (an active rotation):

(8) $$P=R_{\text{rot}}^{*}P_{0}R_{\text{rot}}=1+\epsilon \left(R_{\text{rot}}^{*}x_{0}R_{\text{rot}}\right)$$

Let us now assume that reference frame R″ has the same orientation as R but R″ origin is offset from R origin. The transformation between both reference frames is a simple translation along a given unitary axis **t** and distance *d*. The unitary axis **t** is parallel to the line connecting the origins of frames R″ and R and is directed from R″ origin toward R origin. In R″ frame, point P has coordinates:

(9) $$X^{\prime \prime }=T_{\text{trans}}X{\bar{T_{\text{trans}}}}^{*}=1+\epsilon (x+dt)$$

where *T*_trans_ is the translation dual quaternion. The conjugate dual quaternion that we use for point transformation (see Bayro-Corrochano, 2003) is ${\bar{T_{\text{trans}}}}^{*}=1-\epsilon (-\frac{d}{2}t)=T_{\text{trans}}$. We can also apply the same reasoning for the active translation of a given point.

The velocity **ẋ** of the 3D physical point P with coordinates **x** in a given reference frame R is represented by the dual quaternion $V\;\dot{=}\;\dot{X}=0+\epsilon \dot{x}$. It can be of interest to compute the velocity of P in the frame R′, which is itself rotating compared to R. In that reference frame R′, the velocity dual quaternion *V*′ is:

(10) $$\begin{array}{l} V^{\prime }=\frac{d(R_{\text{rot}}XR_{\text{rot}}^{*})}{dt}\\ \;=\dot{R_{\text{rot}}}XR_{\text{rot}}^{*}+R_{\text{rot}}XR_{\text{rot}}^{\dot{*}}+R_{\text{rot}}\dot{X}R_{\text{rot}}^{*} \end{array}$$

Using relationship (4), expression (10) may be developed:

$$V^{\prime }=R_{\text{rot}}\left(\frac{1}{2}\left(\Omega_{rot}X-X\Omega_{rot}\right)+V\right)R_{\text{rot}}^{*}$$

We can observe that if frame R′ does not move compared to frame R, Ω_**rot**_ = **0**, then the only difference is that *V*′ has its coordinates expressed in the R′ frame instead of the R frame.

From another point of view, we may want to know the velocity *Ṗ* of a mobile point *P* during a rotation *R*_rot_(*t*) applied to a reference position *P*_0_ = 1 + ϵ**x**_0_ with reference velocity *Ṗ*_0_ in a given reference frame R (an active rotation):

$$\begin{array}{l} \dot{P}=\frac{d(R_{\text{rot}}^{*}P_{0}R_{\text{rot}})}{dt}\\ \;=\frac{1}{2}\left(P\Omega_{rot}-\Omega_{rot}P\right)+R_{\text{rot}}^{*}{\dot{P}}_{0}R_{\text{rot}} \end{array}$$

Let us now compute the velocity *V*″ of point P in the reference frame R″ that is translated with respect to R. The transformation between both reference frames is a simple translation motion along a given unitary axis **t** and distance *d*(*t*). In R″ frame, the velocity of point P is:

(11) $$\begin{array}{l} V^{\prime \prime }=\frac{d(T_{\text{trans}}XT_{\text{trans}})}{dt}\\ \;=\underset{=\epsilon \dot{d}t}{\underset{︸}{\dot{T_{\text{trans}}}XT_{\text{trans}}+T_{\text{trans}}X\dot{T_{\text{trans}}}}}+\underset{=\dot{X}}{\underset{︸}{T_{\text{trans}}\dot{X}T_{\text{trans}}}}\\ \;=0+\epsilon (\dot{x}+\dot{d}t) \end{array}$$

For some applications, we may be interested in applying the kinematic operators to lines instead of points. For example, we may be interested in the orientation of an end-effector (in addition to the position) and/or the way this orientation changes with time. Lines provide a useful way to answer those questions. An example will be described in the applications. The operations are exactly identical to those described for point transformations except that another dual quaternion conjugate is used: the quaternion conjugate, A^*^, instead of the mixed conjugate, $\bar{A^{*}}$ (see Equations 7, 9). This slight difference is due to the way that points and lines are encoded with dual quaternions (Bayro-Corrochano, 2003).

### 2.5. Screw motion

Before moving to the applications, we describe a last (less known) kinematic operator: a screw displacement, or screw motion. First we explain what a screw motion is and how this kinematic operation is encoded with dual quaternions. Then we derive the screw motion velocity. Finally we show how screw motion can be applied easily to both points and lines.

The reader should be aware that the screw motion is not a revolutionary kinematic operator, but it is a simple alternative, providing a single dual quaternion directly for an operation which can be also obtained by combining a translation along and a rotation around an axis line which is offset from the origin. Whatever the approach chosen by the user, the final result for your application will be the same. However, screw motion dual quaternions provide a more compact way to write the kinematics for this type of movement.

#### 2.5.1. Screw motion definition

A screw motion describes a rotation of angle θ about a line whose direction is specified by a unitary axis **n** and whose location can be described by an arbitrarily chosen point on the line, with coordinates **a**, followed/combined by a translation of distance *d* along this axis **n**. **a** is an arbitrary point of the line and this line does not necessarily go through the origin, such that the screw motion formalism also can be used to describe rotations about eccentric points—in contrast to quaternions and rotation matrices, which can only characterize rotations about the origin. A screw motion can be represented by the following dual quaternion:

(12) M​=​(​cosθ2+n sinθ2​)​+ϵ​(​−d2sinθ2+nd2cosθ2+(a→∧n→)sinθ2​)

Note that the quantity $\vec{a}\wedge \vec{n}$ is independent of the vector **a** we choose on the line to describe the line location. Equation (12) is derived (see Daniilidis, 1999; Bayro-Corrochano, 2003) by starting from the rotation quaternion *R* describing a rotation of angle θ around the unitary axis **n**. However, as previously noted, the quaternion formulation implicitly assumes that the rotation axis line passes through the reference frame origin. If the rotation line passes through a point with coordinates **a**, a general expression for the rotation dual quaternion whose axis is offset by **a**, *R*_*T*_, is obtained by applying a translation operator $T_{L}=1+\epsilon \frac{a}{2}$ to the quaternion *R*, by left-multiplying it by the conjugate of *T*_*L*_ and right-multiply it by *T*_*L*_. It is quite similar to the translation operation that we apply to lines (instead of rotation dual quaternion here) and that we described in section 2.4 :

RT=TL*RTL    =(1−ϵa2)(cosθ2+n sinθ2)(1+ϵa2)    =(cosθ2+n sinθ2)+ϵ((a→∧n→)sinθ2)

The dual quaternion *R*_*T*_ describes the same rotation but takes the offset between the reference frame origin and the line into account. *R*_*T*_ is a transformation of *R* and therefore *T*_*L*_ had to be applied from both sides. To obtain the screw motion dual quaternion *M*, we need to combine a translation $T=1+\epsilon \frac{d}{2}n$ along the line axis to the rotation *R*_*T*_ (here we combine operators instead of transforming one operator, so there is only a simple left-multiplication):

$$\begin{array}{l} M=TR_{T}=\left(\cos\frac{\theta }{2}+n\;\sin\frac{\theta }{2}\right)\\ \;+\epsilon \left(-\frac{d}{2}\sin\frac{\theta }{2}+n\frac{d}{2}\cos\frac{\theta }{2}+(\vec{a}\wedge \vec{n})\sin\frac{\theta }{2}\right) \end{array}$$

#### 2.5.2. Screw motion velocity

The screw motion velocity dual quaternion, *Ṁ*, is derived from the screw motion dual quaternion expression, *M*, in Equation (12). Indeed, a screw motion can be decomposed into a translational part and a rotational part, as shown in section 2.5.1. We have:

$$\begin{array}{l} M=TR_{T}\\ \;=TT_{L}^{*}RT_{L} \end{array}$$

Therefore,

(13) $$\begin{array}{l} \dot{M}=\dot{T}T_{L}^{*}RT_{L}+T{\dot{T}}_{L}^{*}RT_{L}+TT_{L}^{*}R{\dot{T}}_{L}+TT_{L}^{*}\dot{R}T_{L}\\ \;=\underset{\text{translationalterm}}{\underset{︸}{\dot{T}R}}+\underset{\text{offsetterm}}{\underset{︸}{{\dot{T}}_{L}^{*}R+R{\dot{T}}_{L}}}+\underset{\text{rotationalterm}}{\underset{︸}{\frac{1}{2}TT_{L}^{*}R\Omega T_{L}}} \end{array}$$

where the second line is obtained through a simple calculation (translation has no effect on a velocity dual quaternion). We can observe the contributions of several terms related to the translational velocity along the screw axis, the translational velocity of the screw axis itself and the rotational velocity term.

Similarly to rotation and translation operations, we may easily apply screw motion operations on points or lines.

#### 2.5.3. Screw motion applied to points

Let us consider now an active screw motion *M* = *R*_*T*_*T* applied to a reference point *P*_0_. The resulting position is then:

(14) $$\begin{array}{l} P={\bar{M}}^{*}P_{0}M\\ \;=T{\bar{R_{T}}}^{*}P_{0}R_{T}T \end{array}$$

If we consider again the active screw motion *M* = *R*_*T*_*T* applied to the reference point *P*_0_ with velocity *Ṗ*_0_, the resulting velocity is then:

$$\begin{array}{l} \dot{P}=\frac{d({\bar{M}}^{*}P_{0}M)}{dt}\\ \;=\dot{{\bar{M}}^{*}}P_{0}M+{\bar{M}}^{*}P_{0}\dot{M}+{\bar{M}}^{*}{\dot{P}}_{0}M \end{array}$$

As will be seen in the applications, we can linearly combine several rotations, translations and screw motions in a compact expression, facilitating the geometrical interpretation and the clarity.

#### 2.5.4. Screw motion applied to lines

Let us describe the final parameters of the line *L* = **n** + ϵ**m** resulting from applying a screw motion described by *M* = *R*_*T*_*T* to a reference line *L*_0_ = **n**_0_ + ϵ**m**_0_. Compared to the expression used for points (see Equation 14), the only slight difference is the use of another conjugate, as previously mentioned (see Bayro-Corrochano, 2003).

$$\begin{array}{l} L=M^{*}L_{0}M\\ \;=T^{*}R_{T}^{*}L_{0}R_{T}T \end{array}$$

Knowing the structure of a line dual quaternion (see Equation 5), we can extract the line parameters **n** and **m**. Since $m=\vec{p}\wedge \vec{n}$ where $\vec{p}$ is any point located on line *L*, we can choose a particular $\vec{p}$ by adding an additional constraint. An example would be to take the point $\vec{p}$ which is the closest to the origin.

We can also compute the line velocity $\dot{L}=\dot{n}+\epsilon \dot{m}$ resulting from a screw motion *M* to a reference line *L*_0_ with velocity ${\dot{L}}_{0}$.

$$\begin{array}{l} \dot{L}=\frac{d(M^{*}L_{0}M)}{dt}\\ \;={\dot{M}}^{*}L_{0}M+M^{*}L_{0}\dot{M}+M^{*}\dot{L_{0}}M \end{array}$$

Again, we can retrieve the parameters **ṅ** and **ṁ**. Having retrieved a particular point **p** of the line, we can extract its velocity (orthogonally to the line orientation **n**).

In the Appendix C, we also describe some useful dual quaternions identities which may be used in the diverse sensorimotor applications that we will present in section 3. In the next section, we discuss the implementation of dual quaternions.

### 2.6. Implementation of dual quaternions

From an implementation perspective, we can write a quaternion $A=a_{0}+i\vec{a}$ as a four-element vector *A*_tab_:

$$A_{\text{tab}}=\left(\begin{array}{c} a_{0}\\ \vec{a} \end{array}\right)=\left(\begin{array}{c} a_{0}\\ a_{1}\\ a_{2}\\ a_{3} \end{array}\right)$$

where $\vec{a}={\left(a_{1}\;a_{2}\;a_{3}\right)}^{T}$ where ^*T*^ is the *transpose* matrix operator. If we consider *N* quaternions simultaneously, we just create a 4 × N matrix where each column is a the vector representation of the corresponding quaternion.

Using this representation, the quaternion multiplication is implemented using matricial operations:

(15) $$A_{\text{tab}}B_{\text{tab}}=\left(\begin{array}{c} a_{0}b_{0}-\vec{a}\cdot \vec{b}\\ a_{0}\vec{b}+b_{0}\vec{a}-\vec{a}\times \vec{b} \end{array}\right)$$

We can also consider *N* simultaneous multiplications of two quaternions. This is achieved by adapting the above formula to matrices instead of vectors. These simultaneous operations are not just for theoretical purposes or generalizations. Indeed, again addressing to the neuroscientists communities, we often have to deal with hundreds or thousands of trials in behavioral experiments. Often, we compare our data to model predictions. In this framework, let us consider that we use quaternions for our model predictions. In this case, it is much faster to transform *N* quaternions (corresponding to *N* trials) simultaneously than to run them individually through a *for* loop.

A dual quaternion $D=D_{0}+\epsilon D_{1}=d_{0}+i\vec{d_{0}}+\epsilon (d_{1}+i\vec{d_{1}})$ can be represented using a 8-dimensional vector *D*_tab_ (which can also be considered as the juxtaposition of two 4-dimensional vectors representing the two quaternion components of the dual quaternion):

$$D_{\text{vec}}=\left(\begin{array}{c} {\left(D_{0}\right)}_{\text{tab}}\\ {\left(D_{1}\right)}_{\text{tab}} \end{array}\right)=\left(\begin{array}{c} d_{0}\\ \vec{d_{0}}\\ d_{1}\\ \vec{d_{1}} \end{array}\right)$$

Again, we can generalize to *N* dual quaternions by using a 8 × *N* matrix. The dual quaternion multiplication is implemented using matricial operations and the quaternion multiplication defined in Equation (15):

$$F_{\text{vec}}=\left(\begin{array}{c} {\left(D_{0}\right)}_{\text{tab}}{\left(E_{0}\right)}_{\text{tab}}\\ {\left(D_{0}\right)}_{\text{tab}}{\left(E_{1}\right)}_{\text{tab}}+{\left(D_{1}\right)}_{\text{tab}}{\left(E_{0}\right)}_{\text{tab}} \end{array}\right)$$

We will not describe how all dual quaternion operators and transformation are implemented, as most of them are easy or just a consequence of the encoding of a general dual quaternion that we just described. However, for the reader who would not like to implement herself/himself all the dual quaternion operations in her/his favorite language, we provide in supplementary materials a dual quaternion toolbox written in MATLAB providing functions implementing all the dual quaternions operations previously mentioned. Furthermore, an example and a read me file are also available. A document listing several quaternion and dual quaternion Matlab toolboxes developed by others is also provided. In this way, the potential user has access to our toolbox but also to others, and therefore he can judge which one is the most suitable for him/herself. We wanted to develop our own Matlab toolbox because the other ones did not gather all the functionalities we needed.

After introducing the dual quaternion algebra in this first part and how it can be applied in kinematics, we now move to the second part of this manuscript. This part describes how dual quaternions and their derivatives can be used to easily describe several applications from sensorimotor control in neuroscience.

## 3. Applications

The dual quaternion algebra is very convenient to express the motion of rigid bodies, especially our body parts. In the following, we describe several applications of this theory. First we describe the reference frame transformations required in the 3D visuomotor transformation for reaching and tracking movements. Then we describe the forward and inverse kinematics problem for a two-link arm focusing only on the end-effector position. For that problem, we will use point transformations. Finally we focus on the forward and inverse kinematics problem for a three-link arm holding a tool whose position and orientation matters. We will use both point and line transformations. One huge advantage of dual quaternions is the fact that we can use them for both point and line transformations, which is not the case with other formalisms (homogeneous matrices are not suitable for line transformation for example, while dual matrices were developed to tackle line transformations and not point transformations).

### 3.1. Reference frame transformation: movement planning

Here we study reference frame transformations in the context of visuomotor transformations. For instance, visually guided arm movements to reach for a seen object. Indeed, the brain has to transform the visual information about the target of interest into a set of motor commands for the arm muscles. To this end, the brain plans the movement ahead (see Shadmehr and Wise, 2005). However, the transformation between retinal information and the spatial motor plan is not trivial (see Blohm and Crawford, 2007), since our eyes and head move relative to the body (and thus relative to the shoulder, insertion point of the arm). For instance, let us consider the motion of a target in space. If the head is rolled toward the left or right shoulder, the projection of the spatial motion onto the retina will be different depending on the head roll angle (Leclercq et al., 2012). Therefore, the brain should take the head roll into account in order to generate an accurate motor plan for the arm. This transformation amounts to expressing the retinal motion into a spatial motion, thus it is a reference frame transformation problem.

In the following we describe two transformations. First we describe how we express the retinal position and motion of a target, e.g., a tennis ball, as a function of the spatial trajectory of this target and the 3D eye-head-shoulder geometry. This transformation is useful for a neuroscientist dealing with behavioral experiments. Indeed, the spatial target position and velocity are often specified by designing the experiment (by choice of the experimenter), while the retinal position and motion can not be measured directly. These signals need to be estimated using the transformation model that we develop in this first part and measured and/or known signals about the 3D eye-head-shoulder kinematics. Then we describe the *inverse* transformation which computes the spatial position and velocity of a target from the knowledge of the retinal position and velocity as well as the 3D eye-head-shoulder geometry. This transformation is important from a neuroscience perspective as it is the transformation that the brain should implement in order to generate a spatially accurate arm movement (see Blohm and Crawford, 2007; Leclercq et al., 2012). Furthermore, using this theoretical transformation, we can easily test hypotheses about the availability, accuracy or precision of a signal (retinal and/or extra-retinal) in the brain. For that last application, we use the retinal position and velocity estimated through the first transformation described above.

In this context, dual quaternions provide a useful tool to express these transformations since they provide a geometrically meaningful way of expressing them, and they are easily implemented using a dual quaternion toolbox (as we provide it in the supplementary materials).

#### 3.1.1. Computing the retinal position and motion

**Figure 1:**
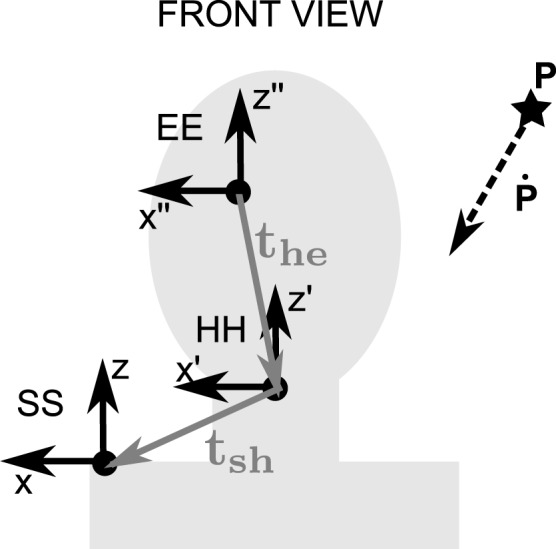
**The picture represents a front view of the eye-head-shoulder system.** The pointing target position (black star) and velocity (dashed arrow) are represented. These position and velocity can be represented in different reference frames, represented on the figure. These reference frames are: SS (shoulder-centered shoulder-fixed), HH (head-centered head-fixed), and EE (eye-centered eye-fixed). The offsets between the rotation centers are also represented: *t*_he_ the head position in eye-centered head-fixed coordinates, and *t*_sh_ the shoulder position in head-centered shoulder-fixed coordinates.

Figure 1 depicts a situation where a subject is confronted with a moving object (e.g., a ball), the target (denoted P), and she/he is going to interact with it (track or catch the target). Here we describe how the retinal position and velocity of the target are expressed as a function of the spatial position and velocity, using the dual quaternion formalism to express the 3D kinematics. In an experiment, we typically specify P position and velocity in a spatial reference frame, for example, the environment reference frame, or the shoulder reference frame in this case since we assume the shoulder remains fixed in the environment. So we need to estimate what the position and the velocity of the projection of P onto the retina are.

In order to compute the P projection trajectory onto the retina, it is obvious that we need to know P kinematics in shoulder (space) coordinates and also what the kinematics of the eye-head-shoulder rigid body system are. In addition to a spatial reference frame, a right-handed orthonormal reference frame is attached to each rigid body (the head, the eye and the shoulder in this example, see Figure 1). Let us assume that P trajectory is known in the shoulder reference frame, *P*^SS^(*t*) = (*P*^SS^_*x*_, *P*^SS^_*y*_, *P*^SS^_*z*_). *P*^SS^(*t*) refers to P position in the **S**houlder-centered **S**houlder-fixed reference frame (*-centered* refers to the reference frame origin while *-fixed* denotes that the orientation of the axes is constant with respect to the specified rigid body) as a function of time.

The first step is to compute P trajectory (position and velocity) in an eye-centered eye-fixed reference frame, denoted *P*^EE^(*t*) and *P*^ĖE^. By combining eye-in-head rotation (*R*_EH_ quaternion), offset between eye and head rotation centers (*T*_HE_ dual quaternion), head-on-shoulder rotation (*R*_HS_ quaternion), and offset between head and shoulder rotation centers (*T*_SH_ dual quaternion), we obtain the following expression for P position in eye-centered eye-fixed coordinates as a function of P position in shoulder-centered shoulder-fixed coordinates (using point transformations, as described in section 2.4; see also Appendix E for the derivation).

(16) $$P^{\text{EE}}=R_{\text{EH}}T_{\text{HE}}R_{\text{HS}}T_{\text{SH}}P^{\text{SS}}T_{\text{SH}}R_{\text{HS}}^{*}T_{\text{HE}}R_{\text{EH}}^{*}$$

where the rotation quaternions are expressed as a function of the rotation angles and unitary axis.

For the P velocity, we differentiate Equation (16). And after a few calculations (see Appendix E), we obtain:

(17) $$\begin{array}{l} \dot{P^{\text{EE}}}=\frac{1}{2}R_{\text{EH}}(\Omega_{EH}P^{\text{EH}}-P^{\text{EH}}\Omega_{EH})R_{\text{EH}}^{*}\\ \;+\;\frac{1}{2}R_{\text{EH}}R_{\text{HS}}(\Omega_{HS}P^{\text{HS}}-P^{\text{HS}}\Omega_{HS})R_{\text{HS}}^{*}R_{\text{EH}}^{*}\\ \;+\;R_{\text{EH}}R_{\text{HS}}\dot{P^{\text{SS}}}R_{\text{HS}}^{*}R_{\text{EH}}^{*} \end{array}$$

where *P*^EH^ (resp.*P*^HS^) is the target position expressed in eye-centered head-fixed (resp. head-centered shoulder-fixed) coordinates. They can be computed, similarly to the expression in Equation (16), as:

$$\begin{array}{l} P^{\text{EH}}=T_{\text{HE}}R_{\text{HS}}T_{\text{SH}}P^{\text{SS}}T_{\text{SH}}R_{\text{HS}}^{*}T_{\text{HE}}\\ P^{\text{HS}}=T_{\text{SH}}P^{\text{SS}}T_{\text{SH}} \end{array}$$

There are three terms in the right side of this expression: the first one depends on the eye-in-head rotational velocity, Ω_**EH**_. The second one depends on the head-on-shoulder rotational velocity, Ω_**HS**_. The third term is related to the target velocity in the shoulder-centered shoulder-fixed reference frame, *P*^ṠS^. When the eye-head-shoulder configuration is static during a trial, only the last term remains. In this case, the only change in velocity is due to the different orientations of the reference frames.

**Figure 2:**
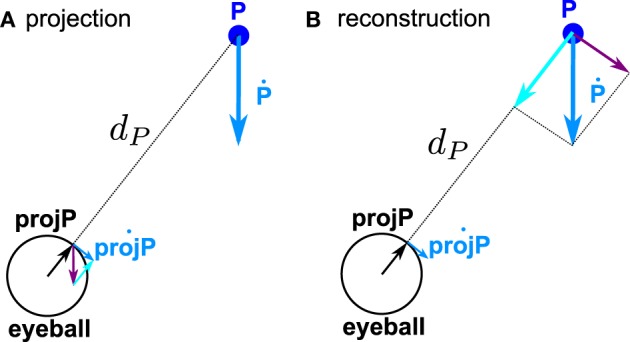
**Geometry of the velocity projection (A) and velocity reconstruction (B). (A)** Projection of P position and velocity onto the eye (actually we plot the reversed retinal projection and velocity projection, to be directly comparable to the spatial position and velocity). References to the reference frame are omitted on the figure for the sake of clarity. The two components which add to obtain $pro\dot{j}P^{EE}$ are represented : 
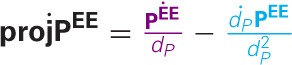
. (**B**) Reconstruction of P position and velocity. The two components which add to obtain **Ṗ^EE^** are represented: 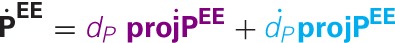.

The first step for computing the retinal position and motion was to express the target position and velocity in a reference frame centered on the eye and fixed to the eye, *P*^EE^(*t*) and *P*^ĖE^. Then the second step is to compute the projection of the target position onto the retina and the resulting velocity of this projection, which is the retinal position and velocity available for further processing in the brain. Computationally, it amounts to project *P*^EE^ = 1 + ϵ **P**^**EE**^ onto a sphere of radius *r*_eye_ (see Figure 2A). For simplicity, we assume *r*_eye_ = 1 (it is just a scaling factor).

$$projP^{\text{EE}}=1+\epsilon \;projP^{EE}$$

where

$$projP^{EE}=\frac{P^{EE}}{\left\|P^{EE}\right\|}=\frac{P^{EE}}{d_{P}}$$

where $d_{P}≜\left\|P^{EE}\right\|$ is the distance of the target (in eye-centered coordinates). The velocity of the projection is computed as follows:

$$\dot{projP^{EE}}=\frac{\dot{P^{EE}}}{d_{P}}-\frac{\dot{d_{P}}P^{EE}}{d_{P}^{2}}$$

The resulting dual quaternion is $pro\dot{j}P^{\text{EE}}=\epsilon \;pro\dot{j}P^{EE}$. Note that if the motion of the target is spherical with the eye as center (isodistance trajectory), then ${\dot{d}}_{P}=0$, and the expression simplifies to $pro\dot{j}P^{EE}=\frac{P^{\dot{E}E}}{d_{P}}$. However, most of the time, the distance is not constant, for example, if the target is moving in a frontoparallel plane. ${\dot{d}}_{P}$ can be expressed as (see Appendix F):

$$\dot{d_{P}}=\frac{P^{EE}\cdot \dot{P^{EE}}}{d_{P}}$$

In this first part, we have computed the retinal target projection vector in eye-centered eye-fixed coordinates from the knowledge of target motion in spatial coordinates. This transformation is useful because we do not know the projection vector in retinal coordinates in advance. Therefore, from an experimental or simulation point of view, this transformation is important. Moreover the above transformation could be useful for the brain if the target is also known to the brain in spatial (or also head) coordinates (for example: auditory target, proprioceptive target in addition to the visual information about the target). Indeed, multisensory integration is carried out using multiple reference frames (McGuire and Sabes, 2009) and therefore auditory (head coordinates) information should be expressed in eye coordinates. This transformation could also be useful if the brain was to implement a forward model, predicting the sensory consequences of the arm motor command, in retinal coordinates.

Now we move to the inverse transformation, which computes the target motion in spatial (or shoulder) coordinates, with the retinal position and velocity as input. This transformation must be carried out by the brain in order to specify a spatially accurate motor plan for the arm (see Blohm and Crawford, 2007; Leclercq et al., 2012).

#### 3.1.2. Computing the spatial position and velocity

Now we describe the model with *projP*^EE^ and $pro\dot{j}P^{\text{EE}}$ as inputs, and *P*^SS^ and *P*^ṠS^ as outputs. These outputs would be used to specify the motor plan (direction, velocity) of an arm tracking movement for example (Leclercq et al., 2012). Using this model, predictions about the motor plan can therefore be easily computed under different hypotheses of incomplete transformation. These hypotheses are made by asking whether extra-retinal signals (3D eye and head positions, 3D eye and head velocities) are available to the brain and whether they are biased or not, and whether they are highly or not much variable (in the framework of bayesian estimation for example).

We start the complete transformation by reversing the above described model. From *projP*^EE^ and $pro\dot{j}P^{\text{EE}}$, we first compute *P*^EE^ and *P*^ĖE^ (see Figure 2B). Theoretically,

$$P^{EE}=d_{P}\;projP^{EE}$$

We observe that *d*_*P*_ is necessary to reconstruct *P*^EE^. It is interesting to consider how the brain estimates *d*_*P*_. Most of the time, our vision is binocular and *d*_*P*_ can be estimated with the use of binocular cues (see Blohm et al., 2008). However, vision is sometimes monocular (e.g., Leclercq et al. (2012) patched one eye in order to have only monocular vision). In this case, no binocular clues are available to estimate target depth. But monocular cues and *a priori* information also help to have an estimation of the target depth, but they are quite reduced for point-like target movements in complete darkness (see Leclercq et al., 2012). A paradigm of tracking in depth would be interesting to test, in monocular and binocular situations. That would allow to differentiate between monocular and binocular clues in order to estimate depth in the context of arm movements.

Theoretically, we also have:

$$\dot{P^{EE}}=d_{P}\;\dot{projP^{EE}}+\dot{d_{P}}projP^{EE}$$

thus ${\dot{d}}_{P}$ also has to be estimated for the estimation of the target velocity in space.

To compute the target position in spatial coordinates, we invert Equation (16), using the fact that the kinematic dual quaternions are unitary and therefore, for any unitary dual quaternion *A*, we have *A*^−1^ = A^*^. Applying that, we have:

$$P^{\text{SS}}=T_{\text{HS}}R_{\text{HS}}^{*}T_{\text{EH}}R_{\text{EH}}^{*}P^{\text{EE}}R_{\text{EH}}T_{\text{EH}}R_{\text{HS}}T_{\text{HS}}$$

where $T_{\text{EH}}=T_{\text{HE}}^{*}=1-\epsilon \;\frac{t_{he}}{2}$ and $T_{\text{HS}}=T_{\text{SH}}^{*}=1-\epsilon \;\frac{t_{sh}}{2}$

For the target velocity in spatial coordinates, we inverse the relationship (17) and obtain:

$$\begin{array}{l} \dot{P^{\text{SS}}}=\frac{1}{2}\left(P^{\text{HS}}\Omega_{HS}-\Omega_{HS}P^{\text{HS}}\right)\\ \;+\frac{1}{2}R_{\text{HS}}^{*}\left(P^{\text{EH}}\Omega_{EH}-\Omega_{EH}P^{\text{EH}}\right)R_{\text{HS}}\\ \;+R_{\text{HS}}^{*}R_{\text{EH}}^{*}\dot{P^{\text{EE}}}R_{\text{EH}}R_{\text{HS}} \end{array}$$

These two transformations are implemented by the brain for the planning of spatially accurate movements, as it has been shown by Blohm and Crawford (2007) and Leclercq et al. (2012). These transformations are useful for the experimenter, in order to estimate the retinal position and motion of a target, and also to make different predictions on the motor plan generated by the brain, depending on hypotheses made about the signals available to the brain (accuracy, precision). However, we believe that dual quaternions are just a tool to easily model this transformation, and they are most likely not used by the brain as a way to implement these transformations. Most likely, these transformations are implemented in a distributed way by a neural network. Blohm et al. (2009) and Blohm (2012) show how these transformations could be achieved by the brain in a distributed way using a biologically inspired artificial neural network. Moreover they relate the properties of the artificial neurons to those of real neurons. Let us mention that dual quaternions are used in these theoretical studies to generate a huge number of input–output pairs (retinal motion as input and spatial motion as output).

### 3.2. Two-link arm movements: forward and inverse kinematics for end-effector position

We will now consider another example of active movements: 3D arm movements. In section 3.2, we focus on the end-effector trajectory of a two-link arm, describing its forward kinematics and the inverse kinematics using the dual quaternion formalism applied to point transformations. In section 3.3, we consider a three-link arm holding a tool (a screwdriver for example) to emphasize the end-effector orientation importance in several everyday life situations. The dual quaternion formalism is again used in order to model the forward and inverse kinematics for the orientation and position of the end-effector, but it is also applied to lines, in order to deal with the orientation transformation.

#### 3.2.1. Forward kinematics for end-effector position

**Figure 3:**
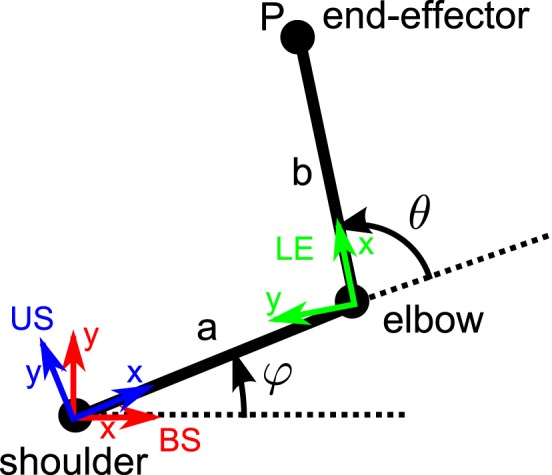
**Two-link arm top view.** The shoulder insertion point, elbow and end-effector location are represented. The different reference frames are also represented: body-fixed shoulder-centered (BS, in red), upper arm -fixed shoulder-centered (US, in blue), and the lower arm -fixed elbow centered (LE, in green). The rotation angles of the shoulder and elbow are respectively denoted φ and θ.

In the following, we consider the forward kinematics of the end-effector position of a two-link arm (see Figure 3). The forward kinematics consists in computing the end-effector position (and sometimes orientation) from the knowledge of the joints kinematics (rotation angles and axes, joint velocities). The advantage of dual quaternions to represent such transformations is the compactness and geometrical significance to express the joint kinematics.

Assuming a length of *a* (resp. *b*) for the upper arm (resp. lower arm), the position *P*_BS_ of the end-effector *P* (*B* for body-fixed coordinates and *S* for **s**houlder-centered coordinates) can be expressed as:

(18) $$P_{\text{BS}}=R_{\text{UB}}^{*}T_{\text{ES}}R_{\text{LU}}^{*}P_{\text{LE}}R_{\text{LU}}T_{\text{ES}}R_{\text{UB}}$$

where *S* stands for shoulder, *U* for upper arm, *E* for elbow and *L* for lower arm. *R*_UB_ represents the upper arm rotation around the shoulder compared to a reference position (rotation angle: φ = 0). *R*_LU_ represents the lower arm rotation around the elbow compared to a reference position (θ = 0). $T_{\text{ES}}=1+\epsilon \frac{1}{2}{\left(a\;0\;0\right)}^{T}$ represents translation dual quaternion associated with the offset between the elbow and the shoulder, in shoulder-centered upper-arm fixed coordinates. *P*_LE_ is the (fixed) position of point *P* in lower-arm fixed elbow-centered coordinates:

$$P_{\text{LE}}=1+\epsilon P_{LE}=1+\epsilon {\left(b\;0\;0\right)}^{T}$$

Note that we can represent the forward kinematics of the end-effector if we know the parameters of the shoulder and elbow rotations (for example, angle and rotation axis), whatever the degrees of freedom.

We can also express the velocity *Ṗ*_BS_ of the end-effector *P*:

(19) $${\dot{P}}_{\text{BS}}=\frac{1}{2}\left(P_{\text{BS}}\Omega_{UB}-\Omega_{UB}P_{\text{BS}}\right)+R_{\text{UB}}^{*}\frac{P_{\text{UE}}\Omega_{LU}-\Omega_{LU}P_{\text{UE}}}{2}R_{\text{UB}}$$

where we obviously assume that **Ṗ**_**LE**_ = **0**. Ω_**UB**_ and Ω_**LU**_ represent the rotational velocities of the upper arm around the shoulder and of the lower arm around the elbow.

#### 3.2.2. Inverse kinematics from end-effector position

Estimating the joint kinematic parameters from the knowledge of the end-effector position and velocity (and sometimes the orientation, see next) is a much more difficult task in general. This process is called inverse kinematics. It can be difficult for several reasons. First, we could solve the inverse kinematics equations (e.g., Equation 19) for the joint kinematic parameters, but theses equations are highly non-linear and no general method exists to solve this problem. For a particular case, it is sometimes possible and advantageous to compute analytical solutions, but it can not always be done. Numerical methods are also widely used to solve this problem, and in the following we will use this approach, which allows us to develop one general method for several different problems. Then, the kinematic system is usually redundant (like the human arm), meaning that there is an infinity of joint configurations that can achieve the prescribed end-effector kinematics. Finally, there are some geometrical configurations in which there are singularities and which tend to complicate the resolution of the inverse kinematics problem. This is a well studied problem in the field of robotics (Klein and Huang, 1983; Cheng and Gupta, 1991; Wang and Chen, 1991; Sciavicco and Siciliano, 1996; Tolani, 2000). Here, we take the two-link arm as an example and develop a methodology from our dual quaternion formalism to compute the inverse kinematics numerically.

The inverse kinematics problem is complicated, especially because the degree of freedom (dof) exceeds the dimension of the end-effector motion (this is called redundancy). For the two-link arm, we consider four degrees of freedom (three at the shoulder and one at the elbow), while the end-effector motion is only 3D. Therefore, there is an infinity of solutions to the problem.

First, we simplify the problem: we assume that the two-link arm moves in the horizontal plane, which reduces the joint dof to 2, yielding a well-posed problem for inverse kinematics. In this case, the velocity dual quaternion representing the 2D velocity of point *P* may be written as (see details in Appendix G.1):

(20) $${\dot{P}}_{\text{BS}}=\underset{J_{\text{DQ}}}{\underset{︸}{\left(\frac{1}{2}\left(P_{\text{BS}}n-nP_{\text{BS}}\right)\;R_{\text{UB}}^{*}\frac{P_{\text{UE}}n-nP_{\text{UE}}}{2}R_{\text{UB}}\right)}}{\left(\dot{\varphi }\;\dot{\theta }\right)}^{T}$$

where **n** is the rotation axis of the shoulder and the elbow (since we only consider planar motions) and where *J*_DQ_ is an array composed of two point velocity dual quaternions (from an implementation perspective, *J*_DQ_ is an 8 × 2 array, see section 2.6). We can build the 2 × 2 jacobian matrix *J* by extracting the 2D vector part of the velocity dual quaternion composing each column of *J*_DQ_ (see Equation 20):

$$J=\left({\left[\frac{1}{2}\left(P_{\text{BS}}n-nP_{\text{BS}}\right)\right]}_{2D\text{vec}}\;{\left[R_{\text{UB}}^{*}\frac{P_{\text{UE}}n-nP_{\text{UE}}}{2}R_{\text{UB}}\right]}_{2D\text{vec}}\right)$$

The dual quaternion equation (20) can then be expressed in a matrix form:

$${\dot{P}}_{BS}=J{\left(\dot{\varphi }\;\dot{\theta }\right)}^{T}$$

where **Ṗ**_**BS**_ is the 2D velocity vector component of the velocity dual quaternion *Ṗ*_BS_. We then compute $\dot{\varphi }$ and $\dot{\theta }$ as a function of **Ṗ**_**BS**_ to solve the inverse kinematics problem, assuming we know the current position of the joints, θ and φ, since the jacobian matrix *J* depends on these parameters: *J* = *J*(θ, φ). Indeed, *J* depends on *P*_BS_, and *P*_BS_ depends on the rotation dual quaternions *R*_UB_ and *R*_LU_ (see Equation 17). Since θ is the rotation angle of *R*_LU_ and φ is the rotation angle of *R*_UB_, it shows that *J* depends on θ and φ. Also, *R*_UB_ and *P*_UE_ depend on φ and appear in the expression of *J*.

However, we can solve for $\dot{\varphi }$ and $\dot{\theta }$ by inverting *J* only if *J* is invertible. It is the case in most geometrical configurations of the two-link arm (indeed, *J* depends on φ and θ, so does the rank of *J*), except when θ = 0 (or θ = 180°), which represents a situation where the upper and lower arms are aligned. In this case, *J* is not invertible (its rank is equal to 1), and we can not apply the above method. This situation can correspond to two cases. If **Ṗ**_**BS**_ has a component parallel to the aligned arm, then there are no solutions for $\dot{\varphi }$ and $\dot{\theta }$. If **Ṗ**_**BS**_ is orthogonal to the aligned arm, then there exists an infinity of solutions (in which case we can express the general form of the solution using the pseudo-inverse formalism, see later in the manuscript).

We notice that the differential velocity relationship linking the joint velocities and end-effector velocity is linear if we assume that θ and φ are known, and that will be the case in the numerical approach we use to solve the inverse kinematics problem. In order to solve the inverse kinematics problem for the joint positions, we can numerically integrate the joint velocities across time, knowing the joint positions at time *t*_0_ (an initial condition is needed). The simplest numerical integration is the Euler method:

(21) $$\begin{array}{l} \theta (t+\Delta t)=\theta (t)+\dot{\theta }(t)\Delta t\\ \varphi (t+\Delta t)=\varphi (t)+\dot{\varphi }(t)\Delta t \end{array}$$

where Δ*t* is the integration step and should not be too large to achieve reasonable accuracy. Then, the new rotation dual quaternions can be updated as a function of θ(*t* + Δ*t*) and φ(*t* + Δ*t*) and we can iterate this process.

Now we want to consider movements in 3D space, not restricted to the plane. In this context, the shoulder has three-dof and the elbow has one-dof. It is clear that there are too many dof compared to the 3D motion of the end-effector, and intuitively many shoulder-elbow configurations will lead to the same end-effector position and velocity. In the following we explain one approach which can be taken to obtain one specific solution for the joints, as well as a general formula to express the set of all possible solutions.

The idea is to start with the dual quaternion expression of the velocity of the end-effector (e.g., Equation 19) and rewrite it in a matrix expression. We move from the dual quaternion representation to the matrix notation because we use tools from the matrix algebra (e.g., the pseudo-inverse matrix) to derive the inverse kinematics results (see below).

The relationship between the end-effector velocity *Ṗ*_BS_ and the rotational velocities of the shoulder, Ω_**UB**_, and the elbow, Ω_**LU**_, is described by Equation (19). Using the fact that: $\frac{1}{2}\left(P_{\text{BS}}\Omega_{UB}-\Omega_{UB}P_{\text{BS}}\right)=-\epsilon \left({\vec{P}}_{\text{BS}}\wedge {\vec{\Omega }}_{\text{UB}}\right)$ (see Appendix G.2) we can show that Equation (19) can be expressed as a linear matrix expression (see Appendix G.3 for the derivation):

(22) $$\underset{J}{\underset{︸}{-\left({\tilde{A}}_{P_{BS}}\;R_{\text{UB}}^{M}{\tilde{A}}_{P_{UE}}\right)}}\left(\begin{array}{c} \Omega_{UB}\\ \Omega_{LU} \end{array}\right)={\dot{P}}_{BS}$$

where **Ṗ**_**BS**_ is the dual bivector part of *Ṗ*_BS_, *R*^*M*^_UB_ is the rotation matrix associated with the rotation dual quaternion *R*_UB_ [see Equation (D.2) in Appendix D for how to compute this rotation matrix] and ${\tilde{A}}_{P_{BS}}$ is the anti-symmetric matrix of rank 2 associated with the cross product: if $\vec{v}=\vec{a}\times \vec{b}$, it can also be written $\vec{v}={\tilde{A}}_{a}\vec{b}$ where:

$${\tilde{A}}_{a}=\left(\begin{array}{c} 0\;-a_{3}\;a_{2}\\ a_{3}\;0\;-a_{1}\\ -a_{2}\;a_{1}\;0 \end{array}\right)$$

where **a** = (*a*_1_ *a*_2_ *a*_3_). Note that this matrix has always rank 2 since for an equation in $\vec{x}$: $\vec{a}\times \vec{x}=\vec{v}$, the component of $\vec{x}$ parallel to $\vec{a}$ can be arbitrary. All the solutions $\vec{x}$ lie on a line.

Coming back to Equation (22), we see that the matrix *J* is of size 3 × 6 and we want to solve for the unknowns Ω_**UB**_ and Ω_**LU**_. Since we consider one-dof at the elbow, the vector Ω_**LU**_ must be aligned with a specific rotation axis **n**_elbow_ = [0 0 1] (in upper arm fixed coordinates) since we assume that the elbow rotates around an axis which is orthogonal to both the lower and upper arms (see also Figure 3 to see why this axis is along the z-axis). This constraint can be written as ${\vec{n}}_{\text{elbow}}\times {\vec{\Omega }}_{\text{LU}}=\vec{0}$. Therefore, we add this constraint to the kinematics equation (22) to obtain:

(23) $$\underset{J}{\underset{︸}{\left(\begin{array}{c} -{\tilde{A}}_{P_{BS}}\;-R_{\text{UB}}^{M}{\tilde{A}}_{P_{UE}}\\ 0\;{\tilde{A}}_{n_{\text{elbow}}} \end{array}\right)}}\left(\begin{array}{c} \Omega_{UB}\\ \Omega_{LU} \end{array}\right)=\left(\begin{array}{c} {\dot{P}}_{BS}\\ 0 \end{array}\right)$$

Now this modified matrix *J* has generally rank 5 since ${\tilde{A}}_{n_{\text{elbow}}}$ has rank 2 (but the rank can be even lower in certain geometrical configurations of the two-link arm, as explained previously with the two-dof two-links arm). Indeed, because we can rotate the two-link arm system around an axis linking the shoulder and the end-effector, without changing the end-effector position and velocity, there is an infinity of solutions (**Ω_UB_, Ω_LU_**) which lie in a one-dimensional manifold, which is mathematically defined as the kernel of *J*, i.e., the set Ker(*J*) = {**x** ∈ ℝ^6^ such that *J***x** = **0**}. Therefore, since there is an infinity of solutions, we need some criterion that may be optimized to choose one optimal solution (according to this criterion), while Equation (23) is a constraint for the optimization problem.

The matrix *J*^−1^ is not defined and thus we choose to use a generalized inverse matrix (which exists for any matrix): the pseudo-inverse [see Klein and Huang (1983) for the use of pseudo-inverse in inverse kinematics], also called the Moore–Penrose inverse. We will denote it by *J*^+^. Therefore, one solution of the redundant system given by Equation (23) is given by:

(24) $$\left(\begin{array}{c} \Omega_{UB}\\ \Omega_{LU} \end{array}\right)=J^{+}{\dot{P}}_{BS}$$

This solution is actually the solution with minimal norm (Klein and Huang, 1983; Sciavicco and Siciliano, 1996), which minimizes the joint velocities in the context of the four-dof two-link arm. The general solution can also be expressed (see Sciavicco and Siciliano, 1996):

$$\left(\begin{array}{c} \Omega_{UB}\\ \Omega_{LU} \end{array}\right)=J^{+}{\dot{P}}_{BS}+\left(I-J^{+}J\right)w$$

where **w** can be any vector in ℝ^6^, and *I* is the identity matrix of order 6 (for this example). In practice, Equation (24) requires that we explicitly compute the pseudo-inverse *J*^+^. The interested reader can refer to Appendix G.4 to see how this can be done.

Once we have a solution for the joint velocities $x=\left(\begin{array}{c} \Omega_{UB}\\ \Omega_{LU} \end{array}\right)$, we can numerically integrate the joint velocities across time, knowing the joints positions at time *t*_0_. Several methods for numerical integration exist (see for example, Atkinson, 1989) and are used in the context of inverse kinematics (see Cheng and Gupta, 1991). Here, we will use the simple Euler method to update the rotation quaternions *R*_UB_ and *R*_LU_. It is a bit more tricky than in the simple two-dof two-link arm (see Equation 21). Indeed, given that $\dot{R}=\frac{1}{2}R\Omega$, we can update the rotation dual quaternion as:

(25) $$R(t+\Delta t)=R(t)+\dot{R}(t)\Delta t$$

but the major problem is that in general *R*(*t* + Δ*t*) is not a rotation quaternion anymore, and we have to normalize it by the norm of *R*(*t* + Δ *t*) to ensure it is a rotation dual quaternion again. From a computational perspective, Funda et al. (1990) showed that the normalization operation is carried out much faster with quaternions than with rotation matrices, which is one of the advantages of using quaternions over rotation matrices. One alternative for the computation is the following. In angular vector notation, the magnitude of the vector represents the rotation angle while the normalized unit vector represents the rotation axis. Using this fact, the rotational displacement between time *t* and *t* + Δ*t* is characterized by the angular vector ΩΔ*t* which is of the form θ**n**. Then, this angular vector can be expressed as a rotation dual quaternion (see Equation 3), Δ*R*. Then,

R(t+Δt)=R(t)ΔR

However, by using such a numerical integration scheme, errors arise since the numerical integration is not perfect. These errors propagate from one iteration to the other and the reconstructed end-effector location (using the forward kinematic model, see Equation 18) will drift from the real end-effector position (see Sciavicco and Siciliano, 1996). In order to avoid this problem, we take the position error between the reconstructed end-effector position, ${\hat{P}}_{BS}$, and the real (or desired) end-effector position, **P**_**BS**_, into account. Actually, we apply the correction scheme described in Sciavicco and Siciliano (1996). The vector error at time *t* is:

$$\begin{array}{l} e(t)=P_{BS}(t)-{\hat{P}}_{BS}(t)\\ \;=P_{BS}(t)-\text{FK}(R_{\text{LU}}(t),R_{\text{UB}}(t)) \end{array}$$

where FK(.) is the forward kinematic function described by Equation (18). We take this error into account by adding a vector term proportional to the error in Equation (23):

(26) $$J\left(\begin{array}{c} \Omega_{UB}(t)\\ \Omega_{LU}(t) \end{array}\right)=\left(\begin{array}{c} {\dot{P}}_{BS}(t)+Ke(t)\\ 0 \end{array}\right)$$

where *K* is a positive definite (usually diagonal) matrix. We can for example choose *K* as the identity matrix multiplied by a factor *k*_1_, which is tuned by the user. Then, we can simply compute a solution using the same pseudo-inverse technique as described above [see Equation (24) to this modified problem].

We described how we deal with the inverse kinematics of the end-effector position of a two-link arm but we can generalize this procedure to a n-link arm. The interested reader can refer to Appendix G.5.

#### 3.2.3. Inverse kinematics: numerical simulation

**Figure 4:**
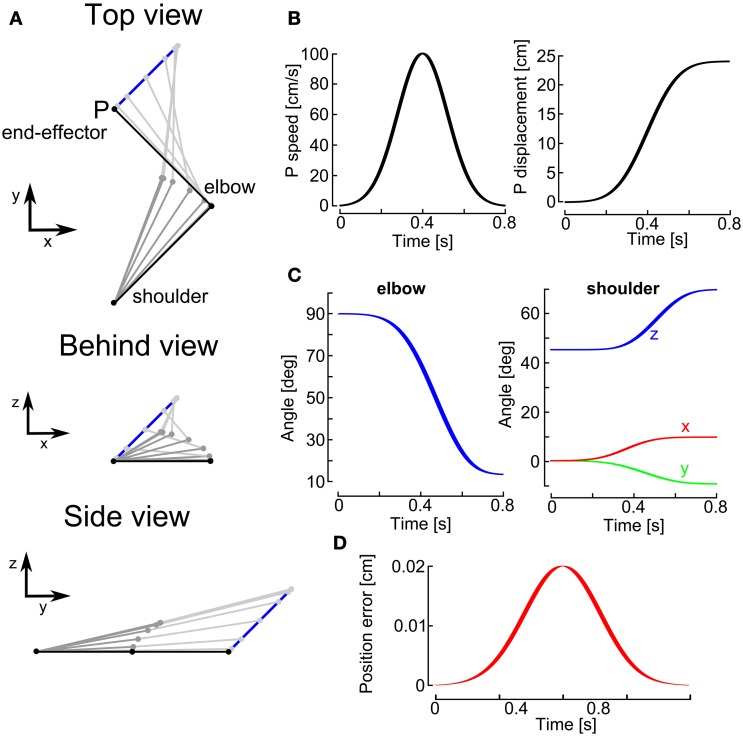
**Numerical simulation example of inverse kinematics of the four-dof two-link arm.** For this simulation, we used *k*_0_ = 1000 and *k*_1_ = 1 (see Appendix G.4) and a simulation step of 1 ms duration. **(A)** Top view (top), behind view (middle), and side view (bottom) of the computed inverse kinematics for the joints. The end-effector (point P) motion in 3D space is described by the blue line. The initial two-link arm configuration is represented in black. Upper arm (dark gray) and lower arm (light gray) are represented at different instants during the end-effector motion. **(B)** The end-effector speed (left) and displacement (right) are described as a function of time: the movement is a typical bell-shaped speed profile lasting 800 ms and with a peak velocity of 100 cm/s. The end-effector displacement is about 25 cm. **(C)** The estimated elbow (left) and shoulder (right) rotation angles are represented as a function of time. For the shoulder, there are three components of rotation: around the x-axis (red line), the y-axis (green line), and the z-axis (blue line). **(D)** Representation of the position error, the norm of the difference between the estimated and actual end-effector position, as a function of time. The maximal error is about 0.02 cm.

Here we test the numerical method for inverse kinematics developed in section 3.2.2 on the four-dof two-link arm. Figure 4 shows one particular example of inverse kinematics for this two-link arm. First, we choose an end-effector motion with a bell-shaped velocity profile and a straight line path in the 3D workspace (see Figure 4B), to mimic hand velocity profiles described in the neuroscience literature (Abend et al., 1982; Atkeson and Hollerbach, 1985). From the subject perspective, the hand is moving from a central position to the right, up and away from the shoulder. The initial configuration of the arm is represented in black (see Figure 4A). From the end-effector motion, we apply the inverse kinematics technique described in the previous section to estimate the joint rotations over time for the movement (Figure 4C).

The estimation accuracy was assessed by computing the norm of the position error, i.e., the difference between the true end-effector position and the estimated end-effector position. The end-effector position was estimated by using the forward kinematic model (see Equation 19) and the estimated joint angles (and therefore rotation quaternions). We can observe (Figure 4D) that the position error was always smaller than 0.02 cm. This position error depends on the value of *k*_1_ (see Equation 26) that we use. For this simulation, we used *k*_1_ = 1000. The larger *k*_1_ the smaller is the position error, because we penalize the position error more with larger values of *k*_1_. But *k*_1_ can not be too large for discrete time systems. If *k*_1_ is too large, the system will be unstable and the error will grow. The *threshold* value for *k*_1_ depends on the simulation step Δ*t* (see Appendix G.6 for details). For our simulation, we used Δ*t* = 1 ms and therefore we could not choose a value for *k*_1_ larger than 2000.

We tested several directions in space for the end-effector motions and our inverse kinematics algorithm was successful in every case, inferring joint angles for the elbow and shoulder. Note that this method does not necessarily reproduce the joints angles observed in human subjects but it yields the solution with the smallest joint angle rotations.

### 3.3. Three-link arm movements: forward and inverse kinematics for end-effector position and orientation

In this section, we describe the forward and inverse kinematics for the end-effector position and orientation of a three-link arm. The main difference compared to section 3.2 is that we perform line transformations (using dual quaternions) to transform the orientation.

For this section, we decided to provide an example where we directly use screw motion to model the joints (shoulder, elbow, and wrist) movements, but we could have kept the same approach as in section 3.2 by alternating rotations and translation (offsets). Similarly, we could have used screw motion dual quaternions as well in section 3.2 for the two-link arm example.

#### 3.3.1. Forward kinematics for end-effector position and orientation

**Figure 5:**
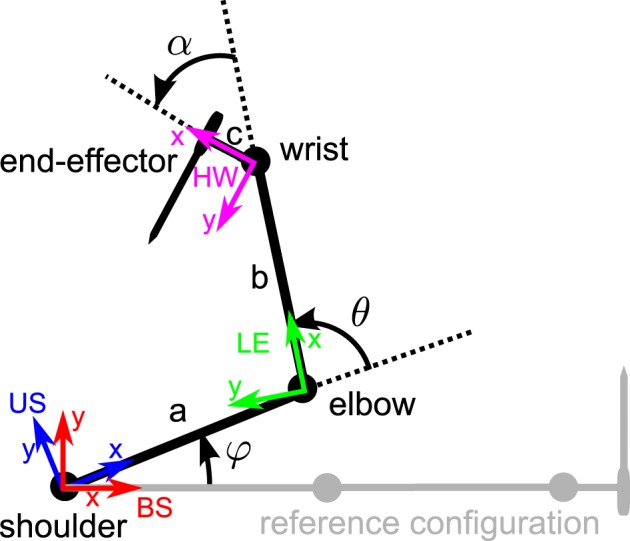
**seven-dof three-link arm top view.** The shoulder insertion point, elbow and wrist are represented as well as the end-effector (a screwdriver) location and orientation. The different reference frames are also represented: body-fixed shoulder-centered (BS, in red), upper arm -fixed shoulder-centered (US, in blue), lower arm -fixed elbow centered (LE, in green), and hand-fixed wrist-centered (HW, in magenta). The rotation angles of the shoulder, elbow, and wrist are respectively denoted φ, θ, and α. The configuration in gray represents the reference configuration.

Here, we describe a seven-dof arm with three links (see Figure 5): the upper and lower arms as well as the wrist. We consider a seven-dof joint model: three for the shoulder, one for the elbow, and three for the wrist. Up to here, we could just apply the methodology described above in section 3.2. However, now we consider an example where the end-effector *orientation* matters. For example, Figure 5 shows that the hand holds a screwdriver, and we are interested in the location and orientation of this end-effector.

In order to describe the end-effector (the screwdriver) orientation (in body-fixed coordinates), we now use the formalism of line dual quaternions (see section 2.3.2). Let $L_{0}=n_{0}+\epsilon ({\vec{r}}_{0}\wedge {\vec{n}}_{0})$ be the line dual quaternion describing the reference line passing through the screwdriver in its reference configuration [see the gray configuration in Figure 5: the arm is fully extended, φ = θ = α = 0° and **n**_0_ = (0 1 0)^*T*^ and **r**_0_ = (*a* + *b* + *c* 0 0)^*T*^]. The forward kinematics describing the end-effector line position, *L*(*t*) = **n**(*t*) + ϵ**m**(*t*), is described by line transformations, using the tools described in the previous sections (again, here we use screw motions but we could have used rotations and translations offsets):

(27) $$\begin{array}{l} L(t)=\left(S_{\text{UB}}^{*}S_{\text{LU}}^{*}S_{\text{HL}}^{*}\right)L_{0}(t)\underset{S_{\text{tot}}}{\underset{︸}{\left(S_{\text{HL}}S_{\text{LU}}S_{\text{UB}}\right)}}\\ \;=S_{\text{tot}}^{*}L_{0}S_{\text{tot}} \end{array}$$

where:

- *S*_UB_ = *R*_UB_ = cos(φ/2) + **n**_**UB**_ sin(φ/2) is a pure rotation quaternion describing the 3D rotation of the upper arm around the shoulder.
- *S*_LU_ = T^*^_ES_*R*_LU_*T*_ES_ is a screw motion dual quaternion describing the 1D rotation of the lower arm around the elbow, whose rotation axis is offset from the origin (the shoulder):
  − *R*_LU_ = cos(θ/2) + **n**_**LU**_ sin(θ/2) is a pure rotation quaternion describing the 3D rotation of the lower arm around the elbow.
  − *T*_ES_ = 1 + ϵ**t**_es_/2 where **t**_es_ = [*a* 0 0]^*T*^ is the offset between the elbow and the shoulder in the reference configuration.
- *S*_HL_ = T^*^_WS_*R*_HL_*T*_WS_ is a screw motion dual quaternion describing the 3D rotation of the hand around the wrist, whose rotation axis is offset from the origin (the shoulder):
  − *R*_HL_ = cos(α/2) + **n**_**HL**_ sin(α/2) is a pure rotation quaternion describing the 3D rotation of the hand around the wrist.
  − *T*_WS_ = 1 + ϵ**t**_ws_/2 where **t**_ws_ = [*a* + *b* 0 0]^*T*^ is the offset between the wrist and the shoulder in the reference configuration.

We can also derive the expression for the end-effector line velocity, $\dot{L}(t)$, deriving the above expressions:

(28) $$\begin{array}{l} \dot{L}(t)=S_{\text{tot}}^{*}{\dot{L}}_{0}(t)S_{\text{tot}}\\ \;+S_{\text{UB}}^{*}S_{\text{LU}}^{*}\left({\dot{S}}_{\text{HL}}^{*}L_{0}S_{\text{HL}}+S_{\text{HL}}^{*}L_{0}{\dot{S}}_{\text{HL}}\right)S_{\text{LU}}S_{\text{UB}}\\ \;+S_{\text{UB}}^{*}\left({\dot{S}}_{\text{LU}}^{*}L_{w}S_{\text{LU}}+S_{\text{LU}}^{*}L_{w}{\dot{S}}_{\text{LU}}\right)S_{\text{UB}}\\ \;+{\dot{S}}_{\text{UB}}^{*}L_{e}S_{\text{UB}}+S_{\text{UB}}^{*}L_{e}{\dot{S}}_{\text{UB}} \end{array}$$

where

- *L*_*w*_ = S^*^_HL_*L*_0_*S*_HL_ is the end-effector line position after applying the wrist rotation to the reference line *L*_0_.
- *L*_*e*_ = S^*^_LU_*L*_*w*_*S*_LU_ is the end-effector line position after applying the elbow rotation to the line *L*_*w*_.

Remembering the description of a screw motion velocity dual quaternion *Ṁ* (see Equation 13) in terms of the pure rotational component, Ω, the offset velocity term *Ṫ*_*L*_ and the translational velocity term along the screw axis *Ṫ*, we can express these screw motion velocities for the seven-dof three-link arm. For each of the screw motion dual quaternions considered here, only the rotational part is moving, in which case *Ṁ* simplifies to $\dot{M}=\frac{1}{2}TT_{L}^{*}R\Omega T_{L}$. Therefore, it is quite easy to compute $\dot{L}(t)=\dot{n}(t)+\epsilon \dot{m}(t)$.

From the line forward kinematics for position and velocity, we can derive the orientation of the end-effector, **n**(*t*), as well as its rate of change, **ṅ**(*t*). The actual position and velocity of the end-effector position is more tricky to obtain. Indeed, we can retrieve the quantities **m**(*t*) and **ṁ**(*t*), but with these quantities, we can only infer the component of the end-effector position orthogonal to the line orientation. And, even if the end-effector position is known, we can not uniquely determine the end-effector velocity component along the line orientation (see also Appendix B). Therefore, in parallel to the line transformation applied to the end-effector line, a position transformation should be applied to the end-effector position. The transformation equations applied to the reference point dual quaternion *P*_0_ = 1 + ϵ**r**_0_ are similar to Equations (27) and (28) except that we use the conjugate definition ${\tilde{S}}^{*}$ instead of S^*^ for point transformation (as described in section 2.4).

#### 3.3.2. Inverse kinematics from end-effector position and orientation

In the case of line transformations, we are also interested in estimating the joint kinematic parameters from the knowledge of the end-effector position and orientation as well as the velocity and orientation rate of change. The problems and the challenges are similar to what we saw before in section 3.2.2 except for the construction of the jacobian matrix *J* which links the joint-velocities to the end-effector velocity and orientation rate of change. For the interested reader, Appendix H describes in detail how to compute this jacobian matrix J for the three-link arm and also generalizes to the n-link arm. Then we can proceed similarly to what is shown in section 3.2.2 to solve the inverse kinematics problem.

## 4. Discussion

In many applications of behavioral neuroscience or vision, there is a need to represent the 3D position and/or orientation of objects as well as their velocity. These objects may be external objects/stimuli that a human subject has to deal with (e.g., the position and orientation of a target object). For example, in order to catch the ball, a rugby player needs to estimate the 3D motion of the ball but also the time course of its orientation, since the ball is non-spherical. This object can also be a body part (e.g., the eye, the head, and the hand), whose position and/or orientation is of interest for the brain to monitor body movements. Dual quaternions provide a convenient compact and geometrically meaningful way to describe either position (through point dual quaternions) or position and orientation (through line dual quaternions). Moreover, dual quaternions provide a way to describe natural geometrical transformations like rotations, translations, or screw motions, and these geometrical transformations can easily be combined together and applied to points or lines. In this paper, we described the useful concepts of the dual quaternion geometric algebra and how dual quaternions can be used to model these transformations. We also described the dual quaternion formalism to cope with velocity: rotational velocity, screw motion velocity, point velocity, and line velocity. Then we applied these concepts to a few examples in behavioral neuroscience: the 3D eye-head-shoulder system reference frame transformation needed for the accurate planning of manual tracking movements. Another example was provided with the forward kinematics for the multi-link arm, either considering the end-effector (the hand) position alone, or considering the end-effector position and/or orientation. Finally we also derived the inverse kinematics of the same multi-link arm from the dual quaternion formalism. In these applications, we do not claim that the brain actually uses dual quaternions to implement these transformations. However, these complex transformations are easily expressed mathematically using dual quaternions, which helps the neuroscience researcher to make predictions, for a theoretical goal or just in designing an experiment.

A main advantage of dual quaternions is that we can combine several rotations and/or translations. Therefore, it is quite easy to compute and write the expression for complex systems. We used that advantage throughout our applications. Another advantage of using quaternions (or dual quaternions) to represent rotations is that it is an efficient way to parameterize rotations, without any singularity (Chou, 1992; Grassia, 1998), which occur when using the classical ways to parameterize rotations (Euler angles for instance). An important advantage of dual quaternions is the compactness in terms of memory requirements: we only need 8 elements to represent them, compared to 12 for homogeneous matrices (Kim and Kumar, 1990; Funda and Paul, 1990a; Aspragathos and Dimitros, 1998; Daniilidis, 1999; Kavan et al., 2008).

Quaternions have been widely used to model 3D eye rotations (Tweed and Vilis, 1987; Hestenes, 1994a; Haslwanter, 1995; Crawford and Guitton, 1997; Bayro-Corrochano, 2003) while dual quaternions have been used less (see Bayro-Corrochano, 2003; Blohm and Crawford, 2007; Leclercq et al., 2012). Here, we described how the dual quaternion formalism can be used to model the multi-body kinematics, which can be useful for modeling purposes in applications like motor planning for eye and arm movements, 3D eye-head gaze shifts, 3D VOR, 3D updating, computing predictions of an inverse or forward internal model.

Dual quaternions have been commonly used in robotics and computer vision, for various purposes. Quaternion parametrization of rotations are used in graphics applications (Grassia, 1998; Tolani, 2000; Azariadis, 2001; Kavan et al., 2008; Ding et al., 2012). Tolani (2000) developed a real-time inverse kinematic technique for anthropomorphic limbs. In Ding et al. (2012), they develop an image processing technique which is built upon a so-called dual quaternion Fourier transform. According to Grassia (1998), quaternions are the best choice to interpolate 3D rotations. Indeed, when we interpolate element by element between two rotations quaternions, it interpolates on the geodesic (shortest) path onto the sphere (Shoemake, 1985; Dam et al., 1998). Dual quaternions are also used for the process of pose estimation, which consists in estimating the position and orientation of an object. In this context, dual quaternions allow to solve simultaneously for both the position and orientation components (Daniilidis, 1999; Bayro-Corrochano et al., 2000) in the context of robotics (hand-eye calibration). Pose estimation is also used with dual quaternions in computer vision (Walker et al., 1991; Phong et al., 1993; Torsello et al., 2011), but with a projective component in addition. In Goddard and Abidi (1998), they used the iterative extended Kalman filter for this purpose of pose estimation in order to deal with uncertainty. Forward kinematics equations for robotic manipulators have been derived with dual quaternions (Kim and Kumar, 1990) and compared with other methods (Aspragathos and Dimitros, 1998). Perez and McCarthy (2004) use dual quaternions for the synthesis of constrained robotic systems where several serial constrained (less than six-dof) manipulators are combined. Inverse kinematics techniques have been developed using dual quaternions for six-dof manipulators (Aydin and Kucuk, 2006; Sariyildiz and Temeltas, 2009) and compared to other methods (Sariyildiz et al., 2011). They are also used in the context of cooperative control of multiple manipulators: several robotic manipulators (Adorno et al., 2010) or a robotic manipulator interacting with a human arm (Adorno et al., 2011). In Pham et al. (2010), they develop a control law using dual quaternion to control simultaneously the position and orientation of a robotic manipulator.

Thus dual quaternions are powerful mathematical constructs that are widely used in robotics and computer vision, and could make important contributions to neuroscience research.

### Conflict of interest statement

The authors declare that the research was conducted in the absence of any commercial or financial relationships that could be construed as a potential conflict of interest.

## Appendix

### A. Multiplication of two quaternions: proof

The multiplication of two quaternions $A=a_{0}+i\vec{a}$ and $B=b_{0}+i\vec{b}$ is a quaternion $C=c_{0}+i\vec{c}$. It is computed as follows:

$$\begin{array}{l} C=AB\\ \;=(a_{0}+i\vec{a})(b_{0}+i\vec{b})\\ \;=a_{0}b_{0}+i(a_{0}\vec{b}+b_{0}\vec{a})-\vec{a}\vec{b}\\ \;=a_{0}b_{0}+i(a_{0}\vec{b}+b_{0}\vec{a})-(\vec{a}\cdot \vec{b}+\vec{a}\wedge \vec{b})\\ \;=\underset{c_{0}}{\underset{︸}{\left(a_{0}b_{0}-\vec{a}\cdot \vec{b}\right)}}\;+\;i\underset{\vec{c}}{\underset{︸}{\left(a_{0}\vec{b}+b_{0}\vec{a}-\vec{a}\times \vec{b}\right)}} \end{array}$$

using the property *i*^2^ = −1 and the expression of the geometric product of two vectors (see Equation 1).

### B. Proof that the line derivative $\dot{L}$ does not depend on the choice of $\vec{p}$

In section 2.3.2, we saw that:

(B.1) $$\dot{L}=\dot{n}+\epsilon \dot{m}$$

(B.2) $$=\dot{n}+\epsilon \left(\dot{\vec{p}}\wedge \vec{n}+\vec{p}\wedge \dot{\vec{n}}\right)$$

One could argue that this line derivative expression depends on the choice of $\vec{p}$ to describe a point on line *L*, and how it changes on this line. We prove in the following that it is not the case. Assume we choose a particular representation: ${\vec{p}}_{1}$ and $\dot{{\vec{p}}_{1}}$. Then the dual component of expression (B.2) is:

$$c_{1}=\dot{\vec{p_{1}}}\wedge \vec{n}+\vec{p_{1}}\wedge \dot{\vec{n}}$$

Assume now that we choose another representation, ${\vec{p}}_{2}$ and $\dot{{\vec{p}}_{2}}$ and that they are linked to the first one in the following way:

$$\begin{array}{l} \vec{p_{2}}=\vec{p_{1}}+x\vec{n}\\ \dot{\vec{p_{2}}}=\dot{\vec{p_{1}}}+\dot{x}\vec{n}+x\dot{\vec{n}} \end{array}$$

Then, the dual component of expression (B.2) is:

$$\begin{array}{l} c_{2}=\dot{\vec{p_{2}}}\wedge \vec{n}+\vec{p_{2}}\wedge \dot{\vec{n}}\\ \;=\left(\dot{\vec{p_{1}}}+\dot{x}\vec{n}+x\dot{\vec{n}}\right)\wedge \vec{n}+\left(\vec{p_{1}}+x\vec{n}\right)\wedge \dot{\vec{n}}\\ \;=\dot{\vec{p_{1}}}\wedge \vec{n}+\dot{x}\underset{=\vec{0}}{\underset{︸}{\vec{n}\wedge \vec{n}}}\;+\;x\dot{\vec{n}}\wedge \vec{n}+\vec{p_{1}}\wedge \dot{\vec{n}}\;+\;x\vec{n}\wedge \dot{\vec{n}}\\ \;=\dot{\vec{p_{1}}}\wedge \vec{n}+\vec{p_{1}}\wedge \dot{\vec{n}} \end{array}$$

which proves that *c*_2_ = *c*_1_ and that the representations are indeed independent of the choice of $\vec{p}$.

### C. Diverse tools and results

#### C.1. Shortest rotation between two unitary vectors

Let us consider two unitary vectors $\vec{a_{1}}$ and $\vec{b_{1}}$. Their quaternion representation (or dual part of their dual quaternion representation) are *A*_1_ = 0 + **a**_**1**_ and *B*_1_ = 0 + **b**_**1**_. We can write the following:

(C.1) $$\begin{array}{l} A_{1}=B_{1}(B_{1}^{-1}A_{1})\\ \;=B_{1}\left(B_{1}^{*}A_{1}\right) \end{array}$$

The expression *B*^*^_1_*A*_1_ can be developed:

(C.2) B1*A1=−b1a1       =b1→a1→       =b1→·a1→+b1→∧a1→       =cosθ+n sinθ

where θ is the rotation angle from vector $\vec{b_{1}}$ toward vector $\vec{b_{1}}$, and $n=\frac{\vec{b_{1}}\wedge \vec{a_{1}}}{\sin\theta }$ is the unitary rotation axis. Together, these two parameters describe the shortest rotation from vector $\vec{b_{1}}$ to vector $\vec{a_{1}}$.

This way of finding the shortest rotation between two unitary vectors can for instance be used to describe the 3D eye rotation obeying Listing's law. As all 3D eye orientations can be described as a rotation from a reference eye position, the Listing's law states that all the possible rotation axes lie in a plane, which is orthogonal to a specific reference eye position, called the primary position. Knowing the primary position and the current gaze position, it is easy to compute the rotation quaternion: *R*_rot_ = cos θ + **n** sin θ describing the rotation angle and rotation axis of the eye, using Equation (C.2).

#### C.2. Shortest screw motion between two lines

Similarly to what is shown in Appendix C.1, we can find the shortest screw motion between two lines *L*_0_ = **n**_**0**_ + ϵ**m**_**0**_ and *L*_1_ = **n**_**1**_ + ϵ**m**_**1**_, where $m_{0}=\vec{r_{0}}\wedge \vec{n_{0}}$ and $m_{1}=\vec{r_{1}}\wedge \vec{n_{1}}$, ${\vec{r}}_{0}$ and ${\vec{r}}_{1}$ being arbitrary points belonging to lines *L*_0_ and *L*_1_. “Shortest” refers to the smallest rotation angle θ and translation distance *d* along the screw axis. Indeed, in 3D space, two lines do not usually intersect. *d* represents the shortest distance between the two lines, and is therefore non-zero when the lines do not intersect. We can write:

$$\begin{array}{l} L_{1}=L_{0}(L_{0}^{-1}L_{1})\\ \;=L_{0}\left(\underset{S}{\underset{︸}{L_{0}^{*}L_{1}}}\right) \end{array}$$

since *L*_0_ and *L*_1_ are unitary dual quaternions. It can be shown that *S* indeed represents the shortest screw motion transformation between *L*_0_ and *L*_1_ and has the following form:

$$\begin{array}{l} S=L_{0}^{*}L_{1}=\cos\theta +\sin\theta n_{S}+\epsilon (-d\sin\theta +dn_{S}\cos\theta \\ \;+(\vec{a_{S}}\wedge \vec{n_{S}})\sin\theta ) \end{array}$$

Therefore, we can compute the angle θ and distance *d* between any two lines in space using this expression.

#### C.3. Screw motion velocity between two moving lines

Following what we did in the Appendix C.2, we can develop the derivatives of the screw motion dual quaternion *S*, to show how to extract quantities like the rotation angle derivative $\dot{\theta }$ or the screw motion translation derivative, $\dot{d}$. Indeed, if we derive *S*, we directly obtain:

$$\begin{array}{l} \dot{S}=-\dot{\theta }\sin\theta +(\dot{\theta }n_{S}\cos\theta +{\dot{n}}_{S}\sin\theta )+\epsilon [-(\dot{d}\sin\theta \\ \;+d\dot{\theta }\cos\theta )+(\dot{d}n_{S}\cos\theta +d{\dot{n}}_{S}\cos\theta -d\dot{\theta }n_{S}\sin\theta \\ \;+\text{​}(\vec{a_{S}}\wedge \vec{n_{S}})\dot{\theta }\cos\theta +(\dot{\vec{a_{S}}}\wedge \vec{n_{S}})\sin\theta +(\vec{a_{S}}\wedge \dot{\vec{n_{S}}})\sin\theta )] \end{array}$$

Although this expression is quite long and difficult to interpret, it is quite easy to retrieve $\dot{\theta }$ and $\dot{d}$ from this dual quaternion, provided we retrieve the parameters of the screw motion *S* first.

### D. Conversion from and to Fick coordinates

In diverse applications, we might want to go from a rotation quaternion expression to Fick coordinates, or vice-versa. Here we briefly describe these mathematical transformations.

Fick coordinates are commonly used in the 3D eye literature (see Haslwanter, 1995), for more comprehensive explanations): they describe a 3D rotation by three successive rotations in a well-defined order. Illustrating the context of a 3D eye position, the first Fick coordinate is a horizontal rotation of θ_*F*_ around a head-fixed axis (θ_*F*_ is positive when the rotation is to the left). Then the second Fick coordinate is a vertical rotation of φ_*F*_ around the inter-aural axis (i.e., the spatial horizontal axis rotated by the first Fick rotation). φ_*F*_ is positive when the rotation is down. Finally, the third Fick coordinate is a torsional rotation of ψ_*F*_ around the line of sight (i.e., the spatial backward–forward axis rotated by the two first Fick rotations). ψ_*F*_ is positive when the rotation is clockwise. All the definitions given above are defined from the point of view of the object being rotated.

A rotation quaternion is described by a rotation angle θ and a rotation axis **n** and is written (see Equation 3):

Rrot=cos(θ2)+n sin(θ2)

#### D.1. Fick coordinates to rotation rotor

Here, we want to compute θ and **n** from Fick coordinates (θ_*F*_, φ_*F*_, ψ_*F*_). First, we compute the 3 × 3 rotation matrix describing the rotation (see Haslwanter, 1995), using **c** and **s** as shortcuts for cos and sin:

(D.1) $$R_{\text{Fick}}=\left(\begin{array}{ccc} c\theta_{F}c\varphi_{F} & c\theta_{F}s\varphi_{F}s\psi_{F}-s\theta_{F}c\psi_{F} & c\theta_{F}s\varphi_{F}c\psi_{F}+s\theta_{F}s\psi_{F}\\ s\theta_{F}c\varphi_{F} & s\theta_{F}s\varphi_{F}s\psi_{F}+c\theta_{F}c\psi_{F} & s\theta_{F}s\varphi_{F}c\psi_{F}-c\theta_{F}s\psi_{F}\\ -s\varphi_{F} & c\varphi_{F}s\psi_{F} & c\varphi_{F}c\psi_{F} \end{array}\right)$$

Then, we need to compute the rotor *R*_rot_ = *q*_0_ + **q** from the rotation matrix. Funda et al. (1990) showed that if the rotation matrix **R** is written:

$$R=\left(\begin{array}{c} a_{11}a_{12}a_{13}\\ a_{21}a_{22}a_{23}\\ a_{31}a_{32}a_{33} \end{array}\right)$$

then, *q*_0_ and **q** = (*q*_1_, *q*_2_, *q*_3_) are given by:

$$\begin{array}{l} q_{0}=\frac{\sqrt{1+a_{11}+a_{22}+a_{33}}}{2}\\ q_{1}=(a_{32}-a_{23})/(4q_{0})\\ q_{2}=(a_{13}-a_{31})/(4q_{0})\\ q_{3}=(a_{21}-a_{12})/(4q_{0}) \end{array}$$

This transformation is for instance needed to reconstruct the 3D eye rotation quaternion, from Fick coordinates provided by a video-based eye tracking system.

#### D.2. Rotation rotor to Fick coordinates

Here we compute the Fick coordinates (θ_*F*_, φ_*F*_, ψ_*F*_) from a rotation quaternion representation Rrot=q0+q=cosθ2+n sinθ2. From *q*_0_ and **q** = (*q*_1_, *q*_2_, *q*_3_), we can retrieve the rotation matrix **R** (see Funda et al., 1990):

(D.2) $$R=\left(\begin{array}{ccc} 1-2q_{2}^{2}-2q_{3}^{2} & 2(q_{1}q_{2}-q_{0}q_{3}) & 2(q_{1}q_{3}+q_{0}q_{2})\\ 2(q_{1}q_{2}+q_{0}q_{3}) & 1-2q_{1}^{2}-2q_{3}^{2} & 2(q_{2}q_{3}-q_{0}q_{1})\\ 2(q_{1}q_{3}-q_{0}q_{2}) & 2(q_{2}q_{3}+q_{0}q_{1}) & 1-2q_{1}^{2}-2q_{2}^{2} \end{array}\right)$$

Then, knowing the structure of **R** in Fick coordinates (Equation D.1), we can identify term by term and extract the Fick parameters (except when there are singularities, in which case one angle can have multiple values).

### E. Derivation of *P*^EE^ and *P*^ĖE^

First we derive step by step the expressions for the pointing target P in eye coordinates. First we show that:

(E.1) $$P^{\text{EE}}=R_{\text{EH}}T_{\text{HE}}R_{\text{HS}}T_{\text{SH}}P^{\text{SS}}T_{\text{SH}}R_{\text{HS}}^{*}T_{\text{HE}}R_{\text{EH}}^{*}$$

Indeed, we obtain this result by applying one operation (rotation or translation) at a time:

$$\begin{array}{l} P^{\text{EE}}=R_{\text{EH}}P^{\text{EH}}R_{\text{EH}}^{*}\\ \;=R_{\text{EH}}T_{\text{HE}}P^{\text{HH}}T_{\text{HE}}R_{\text{EH}}^{*}\\ \;=R_{\text{EH}}T_{\text{HE}}R_{\text{HS}}P^{\text{HS}}R_{\text{HS}}^{*}T_{\text{HE}}R_{\text{EH}}^{*}\\ \;=R_{\text{EH}}T_{\text{HE}}R_{\text{HS}}T_{\text{SH}}P^{\text{SS}}T_{\text{SH}}R_{\text{HS}}^{*}T_{\text{HE}}R_{\text{EH}}^{*} \end{array}$$

where *P*^EH^, *P*^HH^, and *P*^HS^ are the coordinates of the pointing target in eye-centered head-fixed coordinates, head-centered head-fixed coordinates, and head-centered shoulder-fixed coordinates.

For the P velocity, we differentiate Equation (E.1). Differentiating the first line, we write:

(E.2) $$\dot{P^{\text{EE}}}=\dot{R_{\text{EH}}}P^{\text{EH}}R_{\text{EH}}^{*}+R_{\text{EH}}P^{\text{EH}}\dot{R_{\text{EH}}^{*}}+R_{\text{EH}}\dot{P^{\text{EH}}}R_{\text{EH}}^{*}$$

Using the rotational velocity operator (Equation 4), Equation (E.2) may be written:

$$\begin{array}{l} \dot{P^{\text{EE}}}=\frac{1}{2}R_{\text{EH}}(\Omega_{EH}P^{\text{EH}}-P^{\text{EH}}\Omega_{EH})R_{\text{EH}}^{*}+R_{\text{EH}}\dot{P^{\text{EH}}}R_{\text{EH}}^{*}\\ \;=R_{\text{EH}}\left(\frac{1}{2}(\Omega_{EH}P^{\text{EH}}-P^{\text{EH}}\Omega_{EH})+\dot{P^{\text{EH}}}\right)R_{\text{EH}}^{*} \end{array}$$

Because *T*_HE_ is time-invariant (the offset between eye and head rotation centers is obviously fixed in a head-fixed reference frame), we have then

$$\dot{P^{\text{EH}}}=\dot{P^{\text{HH}}}$$

Similarly to Equation (E.2), we have:

(E.3) $$\begin{array}{l} \dot{P^{\text{HH}}}=\dot{R_{\text{HS}}}P^{\text{HS}}R_{\text{HS}}^{*}+R_{\text{HS}}P^{\text{HS}}\dot{R_{\text{HS}}^{*}}+R_{\text{HS}}\dot{P^{\text{HS}}}R_{\text{HS}}^{*}\\ \;=R_{\text{HS}}\left(\frac{1}{2}(\Omega_{HS}P^{\text{HS}}-P^{\text{HS}}\Omega_{HS})+\dot{P^{\text{HS}}}\right)R_{\text{HS}}^{*} \end{array}$$

Finally, we assume *T*_HS_ is time-invariant. Thus,

$$\dot{P^{\text{HS}}}=\dot{P^{\text{SS}}}$$

The final expression for P velocity in an eye-centered eye-fixed reference frame is therefore:

(E.4) $$\begin{array}{l} \dot{P^{\text{EE}}}=R_{\text{EH}}[\frac{1}{2}(\Omega_{EH}P^{\text{EH}}-P^{\text{EH}}\Omega_{EH})\\ \;+\;R_{\text{HS}}\left(\frac{1}{2}(\Omega_{HS}P^{\text{HS}}-P^{\text{HS}}\Omega_{HS})+\dot{P^{\text{SS}}}\right)R_{\text{HS}}^{*}]R_{\text{EH}}^{*}\\ \;=\frac{1}{2}R_{\text{EH}}(\Omega_{EH}P^{\text{EH}}-P^{\text{EH}}\Omega_{EH})R_{\text{EH}}^{*}\\ \;+\;\frac{1}{2}R_{\text{EH}}R_{\text{HS}}(\Omega_{HS}P^{\text{HS}}-P^{\text{HS}}\Omega_{HS})R_{\text{HS}}^{*}R_{\text{EH}}^{*}\\ \;+\;R_{\text{EH}}R_{\text{HS}}\dot{P^{\text{SS}}}R_{\text{HS}}^{*}R_{\text{EH}}^{*} \end{array}$$

### F. Proof of Ḋ_*P*_ expression

Here we compute the expression of ${\dot{d}}_{P}$, which is the rate of change of the distance between the eye and the target *P*. Without loss of generality, we can write:

$$P^{\text{EE}}=1+\epsilon \left(x(t)\;y(t)\;z(t)\right)$$

Therefore,

$$\dot{P^{\text{EE}}}=0+\epsilon \left(\dot{x}(t)\;\dot{y}(t)\;\dot{z}(t)\right)$$

The distance between the eye and the target *P*, denoted *d*_*P*_, is computed from the expression of *P*^EE^:

$$d_{P}=\sqrt{x^{2}+y^{2}+z^{2}}$$

Then, ${\dot{d}}_{P}$ can be developed:

$$\begin{array}{l} \dot{d_{P}}=\frac{x\dot{x}+y\dot{y}+z\dot{z}}{\sqrt{x^{2}+y^{2}+z^{2}}}\\ \;=\frac{P^{EE}\cdot {\dot{P}}_{EE}}{d_{P}} \end{array}$$

### G. Forward and inverse kinematics: derivation of results

#### G.1. Computation of the 2D velocity of the end-effector position

We show that:

$$\dot{P_{\text{BS}}}=\underset{J_{\text{DQ}}}{\underset{︸}{\left(\frac{1}{2}\left(P_{\text{BS}}n-nP_{\text{BS}}\right)\;R_{\text{UB}}^{*}\frac{P_{\text{UE}}n-nP_{\text{UE}}}{2}R_{\text{UB}}\right)}}{\left(\dot{\varphi }\;\dot{\theta }\right)}^{T}$$

Indeed,

$$\begin{array}{l} \dot{P_{\text{BS}}}=\frac{1}{2}\left(P_{\text{BS}}n\dot{\varphi }-\dot{\varphi }nP_{\text{BS}}\right)+R_{\text{UB}}^{*}\frac{P_{\text{UE}}n\dot{\theta }-\dot{\theta }nP_{\text{UE}}}{2}R_{\text{UB}}\\ \;=\left[\frac{1}{2}\left(P_{\text{BS}}n-nP_{\text{BS}}\right)\right]\dot{\varphi }+\left[R_{\text{UB}}^{*}\frac{P_{\text{UE}}n-nP_{\text{UE}}}{2}R_{\text{UB}}\right]\dot{\theta }\\ \;=\underset{J_{\text{DQ}}}{\underset{︸}{\left(\frac{1}{2}\left(P_{\text{BS}}n-nP_{\text{BS}}\right)\;R_{\text{UB}}^{*}\frac{P_{\text{UE}}n-nP_{\text{UE}}}{2}R_{\text{UB}}\right)}}{\left(\dot{\varphi }\;\dot{\theta }\right)}^{T} \end{array}$$

where **n** is the rotation axis of the shoulder and the elbow (since we only consider planar motions)

#### G.2. Proof that $\frac{1}{2}\left(P_{\text{BS}}\Omega_{UB}-\Omega_{UB}P_{\text{BS}}\right)=-\epsilon \left({\vec{P}}_{\text{BS}}\wedge {\vec{\Omega }}_{\text{UB}}\right)$

First, using the definition of a point position dual quaternion *P*_BS_ = 1 + ϵ**P**_**BS**_, we develop the left side of this equation:

$$\begin{array}{l} \frac{1}{2}\left(P_{\text{BS}}\Omega_{UB}-\Omega_{UB}P_{\text{BS}}\right)=\frac{1}{2}\left[\left(1+\epsilon P_{BS})\Omega_{UB}-\Omega_{UB}(1+\epsilon P_{BS}\right)\right]\\ \;=\epsilon \frac{1}{2}\left(P_{BS}\Omega_{UB}-\Omega_{UB}P_{BS}\right) \end{array}$$

Then, let us compute the quaternion multiplications of the two bivectors **P**_**BS**_ and Ω_**UB**_, using the quaternion properties developed in section 2.1:

$$\begin{array}{l} P_{BS}\Omega_{UB}=\left(i{\vec{P}}_{\text{BS}}\right)\left(i{\vec{\Omega }}_{\text{UB}}\right)\\ \;=-{\vec{P}}_{\text{BS}}{\vec{\Omega }}_{\text{UB}}\\ \;=-{\vec{P}}_{\text{BS}}\cdot {\vec{\Omega }}_{\text{UB}}-{\vec{P}}_{\text{BS}}\wedge {\vec{\Omega }}_{\text{UB}} \end{array}$$

Similarly, we have:

$$\Omega_{UB}P_{BS}=-{\vec{\Omega }}_{\text{UB}}\cdot {\vec{P}}_{\text{BS}}-{\vec{\Omega }}_{\text{UB}}\wedge {\vec{P}}_{\text{BS}}$$

Therefore,

$$\begin{array}{l} \frac{1}{2}\left(P_{\text{BS}}\Omega_{UB}-\Omega_{UB}P_{\text{BS}}\right)=\epsilon \frac{1}{2}\left(P_{BS}\Omega_{UB}-\Omega_{UB}P_{BS}\right)\\ \;=\epsilon \frac{1}{2}\left(-{\vec{P}}_{\text{BS}}\wedge {\vec{\Omega }}_{\text{UB}}-(-{\vec{\Omega }}_{\text{UB}}\wedge {\vec{P}}_{\text{BS}})\right)\\ \;=\epsilon \frac{1}{2}\left(-{\vec{P}}_{\text{BS}}\wedge {\vec{\Omega }}_{\text{UB}}-({\vec{P}}_{\text{BS}}\wedge {\vec{\Omega }}_{\text{UB}})\right)\\ \;=-\epsilon \left({\vec{P}}_{\text{BS}}\wedge {\vec{\Omega }}_{\text{UB}}\right) \end{array}$$

#### G.3. Derivation of the matrix expression for Ṗ_BS_

Here, we start from Equation (19) which expresses the velocity of the point end-effector *P* as a function of the rotational velocities of the shoulder and elbow, using the dual quaternion formalism. As a reminder, this equation is:

(G.1) $$\begin{array}{l} {\dot{P}}_{\text{BS}}=\frac{1}{2}\left(P_{\text{BS}}\Omega_{UB}-\Omega_{UB}P_{\text{BS}}\right)\\ \;+R_{\text{UB}}^{*}\frac{P_{\text{UE}}\Omega_{LU}-\Omega_{LU}P_{\text{UE}}}{2}R_{\text{UB}} \end{array}$$

where *P*_UE_ is the position dual quaternion of point *P* in an **u**pper-arm fixed **e**lbow-centered reference frame:

$$P_{\text{UE}}=R_{\text{LU}}^{*}P_{\text{LE}}R_{\text{LU}}$$

Using the relationship derived in Appendix G.2,

$$\frac{1}{2}\left(P_{\text{BS}}\Omega_{UB}-\Omega_{UB}P_{\text{BS}}\right)=-\epsilon \left({\vec{P}}_{\text{BS}}\wedge {\vec{\Omega }}_{\text{UB}}\right)$$

we can develop Equation (G.1) as:

$$\begin{array}{l} {\dot{P}}_{\text{BS}}=\frac{1}{2}\left(P_{\text{BS}}\Omega_{UB}-\Omega_{UB}P_{\text{BS}}\right)+R_{\text{UB}}^{*}\frac{P_{\text{UE}}\Omega_{LU}-\Omega_{LU}P_{\text{UE}}}{2}R_{\text{UB}}\\ \;=-\epsilon \left({\vec{P}}_{\text{BS}}\wedge {\vec{\Omega }}_{\text{UB}}\right)+R_{\text{UB}}^{*}\left[-\epsilon \left({\vec{P}}_{\text{UE}}\wedge {\vec{\Omega }}_{\text{LU}}\right)\right]R_{\text{UB}}\\ \;=-\epsilon \left[\left({\vec{P}}_{\text{BS}}\wedge {\vec{\Omega }}_{\text{UB}}\right)+R_{\text{UB}}^{*}\left({\vec{P}}_{\text{UE}}\wedge {\vec{\Omega }}_{\text{LU}}\right)R_{\text{UB}}\right]\\ \;=-\epsilon \left[i\left({\vec{P}}_{\text{BS}}\times {\vec{\Omega }}_{\text{UB}}\right)+R_{\text{UB}}^{*}i\left({\vec{P}}_{\text{UE}}\times {\vec{\Omega }}_{\text{LU}}\right)R_{\text{UB}}\right] \end{array}$$

Using the rotation matrix *R*^*M*^ corresponding to the rotation dual quaternion *R* [see Equation (D.2) in Appendix D for how to compute this rotation matrix from the rotation dual quaternion], the rotation of a point *P*, written as *P*′ = *R*^*^*PR* in dual quaternion formalism, is written as ${\vec{P}}^{\prime }=R^{M}\vec{P}$ in matrix notation. Furthermore, a cross product of two vectors $\vec{v}=\vec{a}\times \vec{b}$ can also be written as $\vec{v}={\tilde{A}}_{a}\vec{b}$ where

$${\tilde{A}}_{a}=\left(\begin{array}{c} 0\;-a_{3}\;a_{2}\\ a_{3}\;0\;-a_{1}\\ -a_{2}\;a_{1}\;0 \end{array}\right)$$

where **a** = (*a*_1_ *a*_2_ *a*_3_). Note that this matrix has always rank 2 since for an equation in $\vec{x}$: $\vec{a}\times \vec{x}=\vec{v}$, the component of $\vec{x}$ parallel to $\vec{a}$ can be arbitrary. All the solutions $\vec{x}$ lie on a line.

Using these two properties, *Ṗ*_BS_ can be developed further and its vector velocity component, **Ṗ**_**BS**_, can be written in matrix form as:

$${\dot{P}}_{BS}=-\left({\tilde{A}}_{P_{BS}}\;R_{\text{UB}}^{M}{\tilde{A}}_{P_{UE}}\right)\left(\begin{array}{c} \Omega_{UB}\\ \Omega_{LU} \end{array}\right)$$

#### G.4. Computation of the pseudo-inverse *J*^+^

In this appendix, we provide a brief tutorial on the computation of the pseudo-inverse *J*^+^ to the interested reader. We also explain how to deal with solutions which are close to singularities.

In practice, Equation (24) requires that we compute explicitly the pseudo-inverse *J*^+^, by applying for example an algorithm based on the svd (singular value decomposition) of *J* [see Klein and Huang (1983), it is also what Matlab does to compute it]. The advantage is that the pseudo-inverse exists whatever the rank *k* of the matrix J which is of size *n* × m, even if *k* < min(*n, m*). However, the computation time is quite large. If the matrix *J* has rank *k* = min(*n, m*), we can solve $Jx=\dot{v}$ (where $\dot{v}$ is the velocity of the end-effector) without explicitly computing the pseudo-inverse (Klein and Huang, 1983). Indeed, in the case where the system is underdetermined, *k* = *m*, *m* < *n*, the pseudo-inverse is:

$$J^{+}=J^{T}{\left(JJ^{T}\right)}^{-1}$$

while if the system is overdetermined, *k* = *n*, *n* < *m*, then:

$$J^{+}={\left(J^{T}J\right)}^{-1}J^{T}$$

Taking advantage of this formulation, if the system is underdetermined, we solve classically for:

(G.2) $$(JJ^{T})w=\dot{v}$$

where **w** is an intermediate variable from which **x** is computed by taking**x** = *J*^*T*^**w**. If the system is overdetermined, then the solution **x** is obtained by solving:

(G.3) $$(J^{T}J)x=J^{T}\dot{v}$$

Using these methods, a solution for **x** is obtained much faster than by explicitly computing the pseudo-inverse *J*^+^. However, these methods do not work properly if there are singularities in the kinematic system. Singularities happen for example when the two links of the arm are aligned. In this case, the rank of the jacobian *J* is diminished by at least 1, and we can no longer apply this technique. Either, we manually remove one linearly dependent row of *J*, or we compute the explicit pseudo-inverse. Moreover, it can be that there are no solutions when a singularity is met. If the two links are aligned and if the velocity of the end-effector has a component aligned with the two links, there are no solutions (see Sciavicco and Siciliano, 1996).

Finally, in the vicinity of a singularity, the joint velocities may become very large. A solution to this last problem can be found by applying the *damped least-squares (DLS) inverse* instead of the pseudo-inverse. It consists in using the term *JJ*^*T*^ + *k*_0_*I* (where *I* is the corresponding identity matrix, and *k*_0_ is a scalar to be chosen) instead of *JJ*^*T*^ in Equation (G.2) or *J*^*T*^*J* + *k*_0_*I* in Equation (G.3). The equations are therefore better conditioned. As *k*_0_ increases, the joint velocities decrease but at the price that they do not obey perfectly the equations $Jx=\dot{v}$.

#### G.5. n-link arm: forward and inverse kinematics of the end-effector position

In the main text, we have introduced the problem of inverse kinematics and its various difficulties through the example of the four-dof two-link arm: how to deal with redundancy, how to deal with singularities, how to deal with imperfect numerical integration. In the following, we describe how to build the jacobian matrix for a general serial manipulator (something like an arm with *n* links). The methodology and the problems are the same but the size of the system is bigger.

The forward kinematic of a n-link arm end-effector position is:

$$\begin{array}{l} P^{N}=\left(R_{N}^{*}T_{N-1}R_{N-1}^{*}\ldots T_{1}R_{1}^{*}\right)P^{0}\left(R_{1}T_{1}\ldots R_{N_{1}}T_{N-1}R_{N}\right)\\ \;=\underset{{\tilde{S}}_{\text{tot}}^{*}}{\underset{︸}{R_{N}^{*}\left(\prod_{i=1}^{N-1}T_{i}R_{i}^{*}\right)}}P^{0}\underset{S_{\text{tot}}}{\underset{︸}{\left(\prod_{i=1}^{N-1}R_{i}T_{i}\right)R_{N}}} \end{array}$$

where *P*^0^ is the end-effector position dual quaternion in the end-effector fixed reference frame, $R_{i}=\cos\frac{\theta_{i}}{2}+n_{i}\sin\frac{\theta_{i}}{2}$ is the rotation quaternion representing the rotation from link *N* − *i* + 1 to link *N* − *i* (link 0 is the inertial base reference frame) in *N* − *i* link-fixed coordinates, and $T_{i}=1+\epsilon \frac{d_{i}}{2}t_{i}$ is the translation from link *N* − *i* + 1 to link *N* − *i* (link 0 is the inertial base reference frame) in *N* − *i* link-fixed coordinates.

Its velocity can be derived:

$$\begin{array}{l} \dot{P^{N}}={\left(\prod_{j=1}^{N}R_{j}\right)}^{*}\dot{P^{0}}\left(\prod_{j=1}^{N}R_{j}\right)\\ \;+\sum_{i=1}^{N-1}\left[{\left(\prod_{j=i+1}^{N}R_{j}\right)}^{*}2\dot{T_{i}}\left(\prod_{j=i+1}^{N}R_{j}\right)\right]\\ \;+\sum_{i=1}^{N}\left[{\left(\prod_{j=i+1}^{N+1}R_{j}\right)}^{*}\frac{P^{i}\Omega_{i}-\Omega_{i}P^{i}}{2}\left(\prod_{j=i+1}^{N+1}R_{j}\right)\right] \end{array}$$

where *R*_*N* + 1_ = 1, which is set in order to avoid writing one additional line for the term of the sum where *i* = *N*, and where

$$P^{i}={\left[\left(\prod_{j=1}^{i-1}R_{j}T_{j}\right)R_{i}\right]}^{*}P^{0}\left[\left(\prod_{j=1}^{i-1}R_{j}T_{j}\right)R_{i}\right]$$

where *Ṫ*_*i*_ is the translation velocity dual quaternion from link *N* − *i* + 1 to link *N* − *i*, Ω_*i*_ is the rotational velocity between link *N* − *i* + 1 to link *N* − *i*, and *Ṗ*^0^ is the end-effector velocity dual quaternion in the end-effector fixed reference frame (most of the time, *Ṗ*^0^ = 0). From this expression, we can build the jacobian *J* and then write the inverse kinematic equations $Jx=\dot{v}$:

$$\left(\prod_{j=1}^{N}R_{N-j+1}^{M}\;\underset{i=1,\ldots ,N-1}{\underset{︸}{\ldots \;\prod_{j=1}^{N-i}R_{N-j+1}^{M}\;\ldots }}\underset{i=1,\ldots ,N}{\underset{︸}{\ldots \;\left(\prod_{j=0}^{N-i}R_{N-j+1}^{M}\right)\left(-{\tilde{A}}_{P^{i}}\right)\;\ldots }}\right)\left(\begin{array}{c} \dot{P^{0}}\\ \vdots \\ \dot{T_{i}}\\ \vdots \\ \Omega_{i}\\ \vdots \end{array}\right)={\dot{P}}^{N}$$

Then, we predict the rotation and translation dual quaternions at time *t* + Δ*t*, *R*_*j*_(*t* + Δ*t*), and *T*_*j*_(*t* + Δ*t*), similarly to Equation (25).

#### G.6. Threshold value for the feedback term in the numerical procedure

As explained in section 3.2.3 where we developed the numerical simulation example, we can take into account the difference between the true end-effector position and the end-effector position estimated through the reconstructed joint angles. To do so, we tune a parameter, *k*_1_. The larger *k*_1_ the smaller is the position error, because we penalize the position error more with larger values of *k*_1_. But *k*_1_ can not be too large for discrete time systems. If *k*_1_ is too large, the system will be unstable and the error will grow. The *threshold* value for *k*_1_ depends on the simulation step Δ*t*. Indeed, the position error is described by a linear differential equation (see Sciavicco and Siciliano, 1996):

$$\dot{e}+Ke=0$$

where **e** is the position error and *K* is the *feedback* matrix, that we fix for the following to be equal to *k*_1_ multiplied by the identity matrix *I* (we penalize the errors in all the dimensions similarly). In discrete time, we have:

$$\frac{e[k+\Delta t]-e[k]}{\Delta t}=-Ke[k]$$

which is reformulated as:

$$\begin{array}{l} e[k+\Delta t]=(I-K\Delta t)e[k]\\ \;=(1-k_{1}\Delta t)Ie[k] \end{array}$$

From this expression, we see that $0<k_{1}<\frac{2}{\Delta t}$ for the system to be stable (in discrete time, the eigenvalues must lie inside the unit imaginary disk in order to be stable).

### H. Inverse kinematics from the end-effector position and orientation

#### H.1. Three-link arm

In order to build the jacobian matrix, we need to transform Equation (28) [where *Ṡ*_*x*_ is the unknown and $\dot{L}(t)$ is known for our inverse kinematics application] into a matrix equation.

First, we need to use the matrix expression for a screw motion transformation applied to a line or a line velocity (which have the same structure). Let us consider a line *L*_0_ = **n**_0_ + ϵ**m**_0_ to which we apply a general screw motion *S*: a rotation of angle θ around a line whose orientation is **s** and that is offset by **a** from the origin (it can be the position of any point on the line), followed by a translation *d* along the line axis **s**. Then, the expression:

(H.1) $$L(t)=S^{*}L_{0}S=n(t)+\epsilon m(t)$$

can be described by the following matrix expression (see proof in Appendix I.1):

(H.2) $$\underset{J}{\underset{︸}{\left(\begin{array}{c} R^{M}0_{3\times 3}\\ {\tilde{A}}_{a}R^{M}-R^{M}{\tilde{A}}_{a}+R^{M}d{\tilde{A}}_{s}R^{M} \end{array}\right)}}\left(\begin{array}{c} n_{0}\\ m_{0} \end{array}\right)=\left(\begin{array}{c} n\\ m \end{array}\right)$$

where *R*^*M*^ is the rotation matrix associated with the rotation quaternion cos(θ/2) + **s** sin(θ/2), **0** is the 3 × 3 matrix whose entries are all 0 and ${\tilde{A}}_{a}$ (resp. ${\tilde{A}}_{s}$) is the anti-symmetric matrix associated with the cross product, **a** × · (resp. **s** × ·).

If we transform *L*_0_ through *N* screw motions *S*_1_, …, *S*_*N*_:

(H.3) $$L(t)={\left(\prod_{i=1}^{N}S_{i}\right)}^{*}L_{0}\left(\prod_{i=1}^{N}S_{i}\right)=n(t)+\epsilon m(t)$$

then the corresponding matrix expression can be computed by combining *N* matrices of the type of *J* in Equation (H.2):

(H.4) $$\prod_{i=1}^{N}J_{N-i+1}\left(\begin{array}{c} n_{0}\\ m_{0} \end{array}\right)=\left(\begin{array}{c} n\\ m \end{array}\right)$$

Let us consider the expression of *L*(*t*) given by Equation (H.1). The line velocity, $\dot{L}(t)$ is given by:

(H.5) $$\begin{array}{l} \dot{L}(t)={\dot{S}}^{*}L_{0}S+S^{*}L_{0}\dot{S}+S^{*}\dot{L_{0}}S\\ \;=\dot{n}(t)+\epsilon \dot{m}(t) \end{array}$$

where ${\dot{L}}_{0}={\dot{n}}_{0}+\epsilon {\dot{m}}_{0}$, *S* is the screw motion operator and *Ṡ* its velocity. Let us write the matrix expression of this equation. The third term of Equation (H.5) consists simply of a screw motion transformation of the same form as shown in Equation (H.1), applied to the line velocity. The sum of the two first terms gathers the velocity component due to the screw motion velocity applied to the line *L*_0_. In Appendix I.2, we show that if we parameterize *S* as *S* = *TT*^*^_*L*_*R**T*_*L*_ as described in sections 2.5.1 and 2.5.2, then we have:

(H.6) $$\begin{array}{l} {\dot{S}}^{*}L_{0}S+S^{*}L_{0}\dot{S}=-R^{*}\left(\epsilon \dot{d}\left({\vec{n}}_{0}\wedge \vec{s}\right)\right)R\\ \;+R^{*}\left(\epsilon \left({\vec{n}}_{0}\wedge \dot{\vec{a}}\right)\right)R-\epsilon \left(\left(R^{*}n_{0}R\right)\wedge \dot{\vec{a}}\right)\\ \;+T_{L}^{*}\left(\vec{\Omega }\wedge {\vec{n}}_{1}+\epsilon \left(\vec{\Omega }\wedge {\vec{m}}_{1}\right)\right)T_{L} \end{array}$$

where **n**_1_ + ϵ**m**_1_ = (*TT*^*^_*L*_*R*)^*^*L*_0_ (*TT*^*^_*L*_*R*). The first term of Equation (H.6) represents the influence of the translation velocity $\dot{d}$ along the screw axis **s** onto the **ṁ** component of $\dot{L}$. The second term represents the influence of the offset velocity **ȧ** between the screw motion line and the origin onto the **ṁ** component again. Finally, the last term represents the influence of the rotational velocity **Ω** around the screw motion line onto both the **ṅ** and **ṁ** components of the line velocity. In matrix terms, Equation (H.6) may be written:

(H.7) $$\underset{K}{\underset{︸}{\left(\begin{array}{c} 0_{3\times 1}0_{3\times 3}-{\tilde{A}}_{n_{1}}\\ -R^{M}\left({\vec{n}}_{0}\wedge \vec{s}\right)R^{M}{\tilde{A}}_{n_{0}}-{\tilde{A}}_{R^{M}n_{0}}-{\tilde{A}}_{a}{\tilde{A}}_{n_{1}}-{\tilde{A}}_{m_{1}} \end{array}\right)}}\text{​}\left(\text{​}\begin{array}{c} \dot{d}\\ \dot{\vec{a}}\\ \vec{\Omega } \end{array}\text{​}\right)\text{​}=\text{​}\left(\text{​}\begin{array}{c} \dot{n}\\ \dot{m} \end{array}\text{​}\right)$$

From all these expressions, we can now build the matrix expression for Equation (28), knowing that for our application, ${\dot{L}}_{0}=0$ (the screwdriver end-effector has no intrinsic velocity in the reference configuration). Therefore we have:

(H.8) $$\underset{A}{\underset{︸}{\left(\begin{array}{c} J_{\text{UB}}J_{\text{LU}}K_{\text{HL}}J_{\text{UB}}K_{\text{LU}}K_{\text{UB}}\\ 0_{3\times 3}{\tilde{A}}_{n_{\text{LU}}}0_{3\times 3} \end{array}\right)}}\left(\begin{array}{c} \Omega_{HL}\\ \Omega_{LU}\\ \Omega_{UB} \end{array}\right)=\left(\begin{array}{c} \dot{n}\\ \dot{m}\\ 0_{3\times 1} \end{array}\right)$$

where *A* is the jacobian matrix of size 9 × 9 of rank 8, which is consistent with the fact that there are seven-dof and six parameters coding for the line *L*. In Equation (H.8), we have:

- *J*_UB_, *J*_LU_, and *J*_HL_ are matrices of the form *J* described in Equation (H.2). *J*_UB_ has only a rotational component, while *J*_LU_ and *J*_HL_ also have an offset component for their rotation axis, **a**_**LU**_ and **a**_**HL**_.
- *K*_UB_, *K*_LU_, and *K*_HL_ are matrices of the form of the last column of matrix *K* in Equation (H.7), since in the seven-dof three-link arm, we only consider rotational velocities. They are therefore of the type: $K_{i}=\left(\begin{array}{c} -{\tilde{A}}_{{n^{\prime }}_{i}}\\ -{\tilde{A}}_{a_{i}}{\tilde{A}}_{{n^{\prime }}_{i}}-{\tilde{A}}_{{m^{\prime }}_{i}} \end{array}\right)$ for *i* = 1, 2, 3 where **n**′_**i**_ and **m**′_**i**_ are obtained as follows:
  $$\begin{array}{l} \left(\begin{array}{c} n_{1}^{\prime }\\ m_{1}^{\prime } \end{array}\right)=\left(\begin{array}{c} R_{\text{UB}}^{M}0_{3\times 3}\\ 0_{3\times 3}R_{\text{UB}}^{M} \end{array}\right)J_{\text{LU}}J_{\text{HL}}\left(\begin{array}{c} n_{0}\\ m_{0} \end{array}\right)\\ \left(\begin{array}{c} n_{2}^{\prime }\\ m_{2}^{\prime } \end{array}\right)=\left(\begin{array}{c} R_{\text{LU}}^{M}0_{3\times 3}\\ -R_{\text{LU}}^{M}{\tilde{A}}_{a_{\text{LU}}}R_{\text{LU}}^{M} \end{array}\right)J_{\text{HL}}\left(\begin{array}{c} n_{0}\\ m_{0} \end{array}\right)\\ \left(\begin{array}{c} n_{3}^{\prime }\\ m_{3}^{\prime } \end{array}\right)=\left(\begin{array}{c} R_{\text{HL}}^{M}0_{3\times 3}\\ -R_{\text{HL}}^{M}{\tilde{A}}_{a_{\text{HL}}}R_{\text{HL}}^{M} \end{array}\right)\left(\begin{array}{c} n_{0}\\ m_{0} \end{array}\right) \end{array}$$

Then we can use the techniques described in the previous sections, using the jacobian matrix to solve the inverse kinematics problem. In the following, we generalize this method to a *n*−link arm whose end-effector orientation matters.

#### H.2. n-link arm

Again the end-effector reference orientation and position in the base frame is modeled by a line *L*_0_ = **n**_0_ + ϵ**m**_0_. The *N*-link motions can each be described by a screw motion *S*_*i*_, *i* = 1, …, *N*. Therefore we have:

$$L(t)={\left(\prod_{i=1}^{N}S_{i}\right)}^{*}L_{0}\left(\prod_{i=1}^{N}S_{i}\right)=n(t)+\epsilon m(t)$$

The line velocity can be expressed as:

$$\begin{array}{l} \dot{L}(t)={\left(\prod_{i=1}^{N}S_{i}\right)}^{*}{\dot{L}}_{0}\left(\prod_{i=1}^{N}S_{i}\right)\\ \;+\sum_{i=1}^{N}\left[{\left(\prod_{j=i+1}^{N}S_{j}\right)}^{*}\left({\dot{S}}_{i}^{*}L_{i-1}S_{i}+S_{i}^{*}L_{i-1}{\dot{L}}_{i}\right)\left(\prod_{j=i+1}^{N}S_{j}\right)\right] \end{array}$$

where $L_{i}={\left(\prod_{j=1}^{i-1}S_{j}\right)}^{*}L_{0}\left(\prod_{j=1}^{i-1}S_{j}\right)$. Now, in matrix terms, using the properties that we derived above, we obtain:

$$\underset{A}{\underset{︸}{\left(\prod_{i=1}^{N}J_{N-i+1}\text{ ​}\overset{i=1,\ldots ,N}{\overset{︷}{\ldots \text{​ }\left(\prod_{j=1}^{N-i+1}J_{N-j+1}\right)K_{i}\text{ ​}\ldots }}\right)}}\left(\begin{array}{c} {\dot{n}}_{0}\\ {\dot{m}}_{0}\\ \vdots \\ \dot{d_{i}}\\ {\dot{a}}_{i}\\ \Omega_{i}\\ \vdots \end{array}\right)=\left(\begin{array}{c} \dot{n}\\ \dot{m} \end{array}\right)$$

where *J*_*i*_ is of the form described by Equation (H.2), using the rotational and translational parameters of link *i*:

$$J_{i}=\left(\begin{array}{cc} R_{i}^{M} & 0_{3\times 3}\\ {\tilde{A}}_{a_{i}}R_{i}^{M}-R_{i}^{M}{\tilde{A}}_{a_{i}}+R_{i}^{M}d_{i}{\tilde{A}}_{s_{i}} & R_{i}^{M} \end{array}\right)$$

and where *K*_*i*_ has the following structure:

$$K_{i}=\left(\begin{array}{ccc} \begin{array}{c} 0_{3\times 1}\\ -R_{i}^{M}(n_{i-1}\times s_{i}) \end{array} & \begin{array}{c} 0_{3\times 3}\\ R_{i}^{M}{\tilde{A}}_{n_{i-1}}-{\tilde{A}}_{R_{i}^{M}n_{i-1}} \end{array} & \begin{array}{c} -{\tilde{A}}_{n_{i}^{\prime }}\\ -{\tilde{A}}_{a_{i}}{\tilde{A}}_{n_{i}^{\prime }}-{\tilde{A}}_{m_{i}^{\prime }} \end{array} \end{array}\right)$$

where:

- $\left(\begin{array}{c} n_{i}\\ m_{i} \end{array}\right)=\left(\prod_{j=1}^{i}J_{j}\right)\left(\begin{array}{c} n_{0}\\ m_{0} \end{array}\right)$
- $\left(\begin{array}{c} {n^{\prime }}_{i}\\ {m^{\prime }}_{i} \end{array}\right)=\left(\begin{array}{cc} R_{i}^{M} & 0_{3\times 3}\\ -R_{i}^{M}{\tilde{A}}_{a_{i}} & R_{i}^{M} \end{array}\right)\left(\prod_{j=1}^{i-1}J_{j}\right)\left(\begin{array}{c} n_{0}\\ m_{0} \end{array}\right)$

We can of course add constraints by including them into the jacobian matrix *A* of Equation (H.9), for example if we want to impose a rotation axis for **Ω**_**i**_. Then, using this jacobian matrix *A*, we can proceed like described before to find a solution for the inverse kinematics problem.

### I. Matrix expressions for screw motion position and velocity transformations

#### I.1. Screw motion transformation

Here we want to express the following line transformation under a matrix form

(I.1) $$L(t)=S^{*}L_{0}S=n(t)+\epsilon m(t)$$

where *L*_0_ = **n**_0_ + ϵ**m**_0_. A screw motion *S* can be parameterized as *S* = *TT*^*^_*L*_*RT*_*L*_ (see section 2.5.1), where *R* is a pure rotation dual quaternion, $T_{L}=1+\epsilon \frac{1}{2}a$ represents the offset of the rotation axis from the origin and *T* = 1 + ϵ*d***s** is the translation along the screw axis. Therefore, we write:

(I.2) $$L(t)=(T_{L}^{*}R^{*}T_{L}T^{*})(n_{0}+\epsilon m_{0})(TT_{L}^{*}RT_{L})$$

We can show that *T*^*^(**n**_0_ + **ϵm**_**0**_)*T* = **n**_**0**_ + ϵ(**m**_**0**_ + *d***s** × **n**_0_). Performing one transformation, rotation or translation, at a time, we can write in matrix form:

$$\begin{array}{l} \left(\begin{array}{c} n(t)\\ m(t) \end{array}\right)=\left(\begin{array}{c} I_{3\times 3}0_{3\times 3}\\ {\tilde{A}}_{a}I_{3\times 3} \end{array}\right)\left(\begin{array}{c} R^{M}0_{3\times 3}\\ 0_{3\times 3}R^{M} \end{array}\right)\left(\begin{array}{c} I_{3\times 3}0_{3\times 3}\\ -{\tilde{A}}_{a}I_{3\times 3} \end{array}\right)\\ \;\left(\begin{array}{c} I_{3\times 3}0_{3\times 3}\\ d{\tilde{A}}_{s}I_{3\times 3} \end{array}\right)\left(\begin{array}{c} n_{0}\\ m_{0} \end{array}\right)\\ \;=\underset{J}{\underset{︸}{\left(\begin{array}{c} R^{M}0_{3\times 3}\\ {\tilde{A}}_{a}R^{M}-R^{M}{\tilde{A}}_{a}+R^{M}d{\tilde{A}}_{s}R^{M} \end{array}\right)}}\left(\begin{array}{c} n_{0}\\ m_{0} \end{array}\right) \end{array}$$

#### I.2. Screw motion velocity

Here we demonstrate that if we parameterize *S* as *S* = *TT*^*^_*L*_*RT*_*L*_ as described in sections 2.5.1 and 2.5.2, then we have:

$$\begin{array}{l} {\dot{S}}^{*}L_{0}S+S^{*}L_{0}\dot{S}=-R^{*}\left(\epsilon \dot{d}\left({\vec{n}}_{0}\wedge \vec{s}\right)\right)R\\ \;+R^{*}\left(\epsilon \left({\vec{n}}_{0}\wedge \dot{\vec{a}}\right)\right)R-\epsilon \left(\left(R^{*}n_{0}R\right)\wedge \dot{\vec{a}}\right)\\ \;+T_{L}^{*}\left(\vec{\Omega }\wedge {\vec{n}}_{1}+\epsilon \left(\vec{\Omega }\wedge {\vec{m}}_{1}\right)\right)T_{L} \end{array}$$

where **n**_**1**_ + ϵ**m**_**1**_ = (*TT*^*^_*L*_*R*)^*^*L*_0_ (TT^*^_*L*_*R*). Indeed, using Equation (13), we have:

$$\begin{array}{l} {\dot{S}}^{*}L_{0}S+S^{*}L_{0}\dot{S}=\underset{\text{translational velocity component}}{\underset{︸}{\left(R^{*}{\dot{T}}^{*})L_{0}(TT_{L}^{*}RT_{L})+(T_{L}^{*}R^{*}T_{L}T^{*})L_{0}(\dot{T}R\right)}}\\ \;+\underset{\text{offset velocity component}}{\underset{︸}{\left(R^{*}{\dot{T}}_{L}+{\dot{T}}_{L}^{*}R^{*})L_{0}(TT_{L}^{*}RT_{L})+(T_{L}^{*}R^{*}T_{L}T^{*})L_{0}({\dot{T}}_{L}^{*}R+R{\dot{T}}_{L}\right)}}\\ \;+\underset{\text{rotational velocity component}}{\underset{︸}{\left(-\frac{1}{2}T_{L}^{*}\Omega R^{*}T_{L}T^{*}\right)L_{0}(TT_{L}^{*}RT_{L})+(T_{L}^{*}R^{*}T_{L}T^{*})L_{0}\left(\frac{1}{2}TT_{L}^{*}R\Omega T_{L}\right)}} \end{array}$$

Using the following properties:

- velocity dual quaternion due to translational velocity: $\underset{\dot{T}}{\underset{︸}{\left(0+\epsilon v\right)}}(n+\epsilon m)=\epsilon \underset{A}{\underset{︸}{vn}}$
- invariance of this velocity dual quaternion due to translations: $\underset{T}{\underset{︸}{\left(1+\epsilon a\right)}}\epsilon A=\epsilon A$
- rotation of this velocity dual quaternion due to translations: *R*^*^(0 + ϵ*A*)*R* = ϵ(*R*^*^*AR*)

The velocity component due to the translational velocity along the screw axis can be developed as follows:

$$\begin{array}{l} \text{translational velocity component}\\ \;=(R^{*}{\dot{T}}^{*})L_{0}(TT_{L}^{*}RT_{L})+(T_{L}^{*}R^{*}T_{L}T^{*})L_{0}(\dot{T}R)\;\\ \;=R^{*}{\dot{T}}^{*}L_{0}R+(T_{L}^{*}R^{*}T_{L}T^{*})L_{0}(\dot{T}R)\;\\ \;=R^{*}\left[-\epsilon \frac{\dot{d}}{2}s(n_{0}+\epsilon m_{0})+(n_{0}+\epsilon m_{0})\epsilon \frac{\dot{d}}{2}s\right]R\;\\ \;=R^{*}\left[\epsilon (-\frac{\dot{d}}{2}sn_{0}+\frac{\dot{d}}{2}n_{0}s\right]R\;\\ \;=-R^{*}\left(\epsilon \dot{d}\left(\vec{n_{0}}\wedge \vec{s}\right)\right)R \end{array}$$

The velocity component due to the screw axis offset velocity can be expressed as:

$$\begin{array}{l} \text{offset velocity component}\\ =(R^{*}{\dot{T}}_{L}+{\dot{T}}_{L}^{*}R^{*})L_{0}(TT_{L}^{*}RT_{L})+(T_{L}^{*}R^{*}T_{L}T^{*})L_{0}({\dot{T}}_{L}^{*}R+R{\dot{T}}_{L})\\ =R^{*}{\dot{T}}_{L}L_{0}R+{\dot{T}}_{L}^{*}R^{*}L_{0}R+R^{*}L_{0}{\dot{T}}_{L}^{*}R+R^{*}L_{0}R{\dot{T}}_{L}\\ =R^{*}({\dot{T}}_{L}L_{0}+L_{0}{\dot{T}}_{L}^{*})R+{\dot{T}}_{L}^{*}(R^{*}L_{0}R)+(R^{*}L_{0}R){\dot{T}}_{L}\\ =R^{*}\left(\epsilon \left({\vec{n}}_{0}\wedge \dot{\vec{a}}\right)\right)R-\epsilon \left(\left(R^{*}n_{0}R\right)\wedge \dot{\vec{a}}\right) \end{array}$$

Finally, the velocity component due to the rotational velocity is:

$$\begin{array}{l} \text{rotational velocity component}\\ \;=\left(-\frac{1}{2}T_{L}^{*}\Omega R^{*}T_{L}T^{*}\right)L_{0}(TT_{L}^{*}RT_{L})\\ \;+(T_{L}^{*}R^{*}T_{L}T^{*})L_{0}\left(\frac{1}{2}TT_{L}^{*}R\Omega T_{L}\right)\\ \;=-\frac{1}{2}T_{L}^{*}\Omega L_{1}T_{L}+\frac{1}{2}T_{L}^{*}L_{1}\Omega T_{L}\\ \;=\frac{1}{2}T_{L}^{*}\left(L_{1}\Omega -\Omega L_{1}\right)T_{L}\\ \;=T_{L}^{*}\left(\vec{\Omega }\wedge {\vec{n}}_{1}+\epsilon \left(\vec{\Omega }\wedge {\vec{m}}_{1}\right)\right)T_{L} \end{array}$$

where *L*_1_ = **n**_1_ + ϵ**m**_1_ = (*R*^*^*T*_*L*_*T*^*^)*L*_0_(*TT*^*^_*L*_*R*).
